# Human amniotic fluid mesenchymal stem cells attenuate pancreatic cancer cell proliferation and tumor growth in an orthotopic xenograft mouse model

**DOI:** 10.1186/s13287-022-02910-3

**Published:** 2022-06-03

**Authors:** Ying-Cheng Chen, Ying-Wei Lan, Shiaw-Min Huang, Chih-Ching Yen, Wei Chen, Wan-Ju Wu, Theresa Staniczek, Kowit-Yu Chong, Chuan-Mu Chen

**Affiliations:** 1 Department of Life Sciences, and Ph.D. Program in Translational Medicine, College of Life Sciences, National Chung Hsing University, Kuo Kuang Rd, Taichung, 402 Taiwan; 2 Bioresource Collection and Research Center, Food Industry Research and Development Institute, Hsinchu, 300 Taiwan; 3 Department of Internal Medicine, China Medical University Hospital, and College of Health Care, China Medical University, Taichung, 404 Taiwan; 4 Division of Pulmonary and Critical Care Medicine, Chia-Yi Christian Hospital, Chiayi, Taiwan; 5 Department of Dermatology, Venereology and Allergology, University Medical Center and Medical Faculty Mannheim, and Center of Excellence in Dermatology, Heidelberg University, 69117 Mannheim, Germany; 6 Department of Medical Biotechnology and Laboratory Science and Division of Biotechnology, College of Medicine, Chang Gung University, Taoyuan, 333 Taiwan; 7 Department of Laboratory Medicine, Chang Gung Memorial Hospital, Linkou, Taoyuan 333 Taiwan; 8 The iEGG and Animal Biotechnology Center, National Chung Hsing University, Taichung, 402 Taiwan; 9 Rong Hsing Research Center for Translational Medicine, Taichung Veterans General Hospital, Taichung, 407 Taiwan

**Keywords:** Amniotic fluid mesenchymal stem cells, Pancreatic cancer, PANC1 cells, Tumorigenicity, Orthotopic xenograft

## Abstract

**Background:**

Pancreatic ductal adenocarcinoma (PDAC) is a malignant cancer and chemotherapy ineffectively treats PDAC, leading to the requirement for alternative tumor-targeted treatment. Human amniotic fluid mesenchymal stem cells (hAFMSCs) have been revealed to suppress tumor growth in various cancers and they are a strong candidate for treating PDAC.

**Methods:**

To evaluate the effects of hAFMSCs on human pancreatic carcinoma cells (PANC1, AsPC1 and BxPC3 cell lines) and the possible mechanism involved, an in vitro cell coculture system was used. A PANC1 orthotopic xenograft mouse model was established and hAFMSCs were injected intravenously at 4 weeks post-xenograft.

**Results:**

An in vitro coculture assay showed that hAFMSCs inhibited PANC1 cell proliferation by inducing S phase cell cycle arrest and increased cell apoptosis in a time-dependent manner. In PANC1 cells, hAFMSCs caused the downregulation of Cyclin A and Cyclin B1 as well as the upregulation of p21 (CDKN1A) at 24 h post coculture. The upregulation of pro-apoptotic factors Caspase-3/-8 and Bax at 24 h post coculture reduced the migration and invasion ability of PANC1 cells through inhibiting the epithelial-mesenchymal transition (EMT) process. In a PANC1 orthotopic xenograft mouse model, a single injection of hAFMSCs showed significant tumor growth inhibition with evidence of the modulation of cell cycle and pro-apoptotic regulatory genes and various genes involved in matrix metallopeptidase 7 (MMP7) signaling-triggered EMT process. Histopathological staining showed lower Ki67 levels in tumors from hAFMSCs-treated mice.

**Conclusions:**

Our data demonstrated that hAFMSCs strongly inhibit PDAC cell proliferation, tumor growth and invasion, possibly by altering cell cycle arrest and MMP7 signaling-triggered EMT.

**Supplementary Information:**

The online version contains supplementary material available at 10.1186/s13287-022-02910-3.

## Background

Pancreatic cancer has been considered as the most lethal human cancer with the lowest 5-year survival rate and the death rate resulting from pancreatic cancer has risen during the past decade among males, further implying that pancreatic ductal adenocarcinoma (PDAC) belongs to the top five leading causes of death of both genders [1]. Merely, 20% of patients have dissectible tumors, for which the optimal 5-year survival rate is 20% [2]. Therefore, pancreatic adenocarcinoma is regarded as one of the most malignant cancers with a late diagnosis [3] and has been considered as a severe disease due to the rapid translocation of tumor cells, with which cancer cells usually migrate from the original location to other locations [4].

In addition to the features of high malignancy and the latency of discovering pancreatic cancer, pervasive therapy resistance leads to a poor patient survival rate. Patients with resectable tumors can undergo pancreaticoduodenectomy (Whipple’s procedure) along with adjuvant therapy to prolong the period of survival [5]. Other patients with unresectable tumors can undergo chemotherapeutic regimens. In past decades, chemotherapies for pancreatic cancer patients have consisted of first-line therapy, including antimetabolite drugs (gemcitabine and fluoropyrimidines) and second-line therapy, including 5-fluorouracil (5-FU) [6, 7] and the FDA approved receptor-targeted drug erlotinib [8, 9]. Since 2011, the combination of regimens has become the gold standard for metastatic cancer, such as 5-FU combined with irinotecan and oxaliplatin (FOLFIRINOX) as well as nab-paclitaxel combined with gemcitabine [10], resulting in tumor response rates of 23% and 31%, respectively [11], compared to the tumor response rate of merely 0–10% when using a single adjuvant [12]. However, these chemotherapeutic drugs have equivalent effects on pancreatic cancer with an overall survival time of 4–6 months [12] and more attention is moving toward cell therapy, especially with mesenchymal stem cells (MSCs).

Stem cells possess the ability to proliferate and differentiate into specialized cells [13] due to their distinctive features of self-renewal and pluripotency [14]. Mesenchymal stem cells (MSCs) expand ex vivo for multiple indefinite passages [15] and are efficient for any mesodermal differentiation [16]. MSCs present disease tropism and immune regulation [17, 18], with many chemokines and cytokines receptors presented on the surface of MSCs [19, 20], which allows MSCs to easily receive tumor-released cytokines, resulting in stem cell homing [21]. Based on these unique traits, MSCs have potential for clinical cell therapy treatment.

Amniotic fluid is a substitute source of embryonic stem cells (ESCs) [22] and human amniotic fluid mesenchymal stem cells (hAFMSCs) have been characterized as highly multipotent with the potential to differentiate into adipocytes, osteocytes, chondrocytes and other cell types [23]. Human amniotic fluid-derived stem cells have been cocultured with human tumor cells, causing decreased viability of tumor cells. In addition, in vivo evidence of tropism to solid tumors [24] makes treatment with hAFMSCs a prospective strategy. Furthermore, hAFMSCs have been suggested to possess an ability similar to bone marrow-derived mesenchymal stem cells (BM-MSCs) [25], which are the most characterized type of stem cells and have been studied in various cancers, including pancreatic cancer [26], with positive outcomes [27].

In this study, we aimed to determine whether transplantation of hAFMSCs results in a better antitumor outcome in terms of tumor growth as well as to investigate the possible mechanism involved in an in vitro cell coculture system and a PDAC orthotopic xenograft mouse model.

## Methods

### Cell lines

hAFMSCs were a gift obtained from Dr. Shiaw-Min Huang at Food Industry Research and Development Institute (Hsinchu, Taiwan), which were isolated from human amniotic fluid as previously described [28]. Cells were received at passage 8 and maintained in minimum essential medium α (α-MEM) (Gibco, Carlsbad, CA, USA) supplemented with 20% fetal bovine serum (FBS; HyClone, Logan, UT, USA), 4 μg/ml bovine fibroblast growth factor-basic (bFGF; PeproTech, Rehovot, Israel), 100 U/ml penicillin, 100 U/ml streptomycin and 25 μg/ml amphotericin B (GeneDireX Inc., Taoyuan, Taiwan). The 10–14th passage of hAFMSCs used in this study for all in vitro and in vivo studies. The purity of hAFMSCs were examined by using BD Stemflow Human MSC Analysis Kit (BD Biosciences). Flow cytometry analysis showed that hAFMSCs were positive for CD73, CD44, CD105, negative for CD11b, CD19, CD34, CD45, HLA-DR expression on the cell surface as described in Huang et al. [28] (Additional file 1: Figure S1). The human pancreatic adenocarcinoma cell lines, PANC1 (BCRC-60284), AsPC1 (BCRC-60494) and BxPC3 (BCRC-60283), were obtained from Bioresource Collection and Research Center (BCRC, Hsinchu, Taiwan). PANC1 cells were cultured in Dulbecco’s modified Eagle’s medium (DMEM) (Gibco) supplemented with 10% FBS, 100 U/ml penicillin, 100 U/ml streptomycin and 25 μg/ml amphotericin B. AsPC1 and BxPC3 cells were cultured in RPMI 1640 medium (Gibco) supplemented with 10% FBS, 4.5 g/l glucose, 10 mM HEPES, 1 mM sodium pyruvate, 100 U/ml penicillin, 100 U/ml streptomycin and 25 μg/ml amphotericin B. All cell lines were incubated at 37 °C in a 5% CO_2_ incubator. (Additional file 2: Table S1)

### Coculturing of pancreatic cancer cells and hAFMSCs cells

PANC1 cells (5.5 × 10^5^), AsPC1 cells (1.6 × 10^6^) and BxPC3 (5.0 × 10^5^) were seeded separately in a 6-well plate, while hAFMSCs (5.5 × 10^5^) seeded in 0.4 μm 6-well hanging inserts (Transwells; Millipore, Burlington, MA, USA) in their original medium overnight for adhesion. The next day before coculturing (herein named Co-PANC1, Co-AsPC1 and Co-BxPC3), cells were washed twice with DPBS and then added the coculture medium (αMEM supplemented with 10% FBS and 1% P/S). Pancreatic cancer cells alone also cultured in coculture medium served as a negative control (herein named PANC1, AsPC1 and BxPC3). After 24 h of incubation, different conditioned pancreatic cancer cells were trypsinized and resuspended for subsequent functional assays.

### Proliferation assay

PANC1 cells alone and Co-PANC1 cells were seeded in 48-well plates at a density of 6 × 10^4^ cells/well and cell numbers were counted in triplicate using a hemocytometer at 24 h and 55 h postincubation. Control (AsPC1 or BxPC3) and Co-culture (Co-AsPC1 or Co-BxPC3) cells were seeded in 6-well plates at a density of 1.6 × 10^6^ cells/well and 5 × 10^5^ cells/well for AsPC1 and BxPC3, respectively. Cell numbers were counted in triplicate using a hemocytometer at 24 h post coculture.

### Colony-forming assay

1 × 10^3^ cells/well of pancreatic cancer cells alone (PANC1, AsPC1 or BxPC3) and coculturing cells (Co-PANC1, Co-AsPC1 or Co-BxPC3) were plated in 12-well plates and the medium was changed every 3 days. After incubation, PANC1 (10 days), AsPC1 (6 days) and BxPC3 (9 days) cell colonies were formed and stained with crystal violet as previously described [29]. Briefly, colonies were washed with phosphate-buffered saline (PBS), fixed with 10% formaldehyde, and then stained with 0.1% crystal violet. Images of each well were obtained by a camera (Canon EOS 77D, Canon, Tokyo, Japan) and digitized images were analyzed in triplicate using ImageJ software.

### Cell cycle assay

PANC1 cells alone and Co-PANC1 cells were harvested for cell cycle analysis using propidium iodide (PI) staining. Trypsinized cells were fixed in 70% ethanol and placed at − 20 °C for 20 min and cell permeablization buffer was then added (0.5% Triton X-100 with 0.05% RNase A in PBS) followed by incubation at 37 °C for 40 min. Cells were then treated with PI staining solution (50 μg/ml) and incubated at 4 °C for 20 min followed by cytometric analysis [30] performed by a BD AccuriTM C6 Plus flow cytometer (BD Biosciences, San Jose, CA, USA).

### Wound-healing assay

Two-well culture inserts (Ibidi GmbH, Munich, Germany) were used. A total of 4 × 10^4^ PANC1 or Co-PANC1 cells were seeded into culture inserts and allowed to adhere for 2 h. Then, the culture inserts were removed and open areas with clear edges were created. Images of the open area were captured using an Olympus IX71 microscope with an AxioCam MRc camera (Olympus, Tokyo, Japan) under bright field microscopy at 40 × magnification at 0 h and 40 h. Images were analyzed in TScratch software [31].

### Migration assay

Transwell cell migration assays were performed using Millicell Hanging Cell Culture Inserts (Millipore). A total of 2 × 10^5^ cells was placed in 8-μm 24-well Transwell inserts with serum-free medium and medium containing 10% FBS was placed in the lower well as a chemoattractant. After 4 h of incubation, cells were stained with crystal violet as previously mentioned. Images of migrating cells were captured with an inverted microscope under bright field with 100 × magnifications and then analyzed using ImageJ.

### Invasion assay

An invasion assay was performed using 8-μm 24-well Transwells (Millipore) that were precoated with diluted Matrigel® (Corning Inc., Corning, NY, USA) and incubated for 4 h at 37 °C. A total of 1.5 × 10^4^ PANC1 and Co-PANC1 cells in serum-free medium were seeded into the precoated Transwells and medium containing 10% FBS was added to the lower wells [32]. After 48 h of incubation, Transwells were subjected to crystal violet staining.

### Quantitative RT-PCR (qRT-PCR)

Total RNA from cells and tissues was isolated using a DNA/RNA/Protein kit (Geneaid, New Taipei, Taiwan) according to the manufacturer’s instructions. cDNA was prepared with a MMLV reverse transcription kit (Protech, Taipei, Taiwan). qRT-PCR was conducted using the 2 × qPCRBIO SyGreen Blue Mix Lo-ROX (PCR Biosystems, London, UK) and a QuantStudio™ 6 Pro Real-Time PCR System (Applied Biosystems Inc., Foster, CA, USA). The gene-specific primer sequences are listed in Addition file 2: Table S1. The following formula was used to determine relative gene expression: ΔΔCt; where Ct indicates the threshold cycle. Relative mRNA expression levels were normalized to the β-actin reference gene [33].

### Western blot analysis

Total protein from cells and tissues was isolated by a DNA/RNA/Protein Extraction Kit (Geneaid). Proteins were separated by SDS-PAGE, transferred to PVDF membranes and stained with Ponceau S for the confirmation of complete transfer. Membranes were blocked with 5% nonfat dry milk in TBS-T (0.1% Tween-20 in TBS buffer) for 1 h. Proteins were then probed overnight at 4 °C with the following antibodies at appropriate dilutions: 1:1000 dilution of N-cadherin (Taiclone, Taipei, Taiwan), 1:1000 dilution of β-catenin (Santa Cruz Biotech. Inc., Santa Cruz, CA, USA), 1:1000 dilution of Cyclin A (Taiclone), 1:1000 dilution of matrix metallopeptidase 7 (MMP7; Taiclone), 1:2000 dilution of Fibronectin and Collagen type I(Proteintech), 1:500 dilution of p21 and Caspase 3 (Arigo), 1:1000 dilution of Caspase-8 (Cell Signaling Technology), 1:1000 dilution of Bax (SAB Signalway Antibody) and 1:25,000 dilution of β-actin (Novus Biologicals, Centennial, CO, USA). After washing with TBS-T, membranes were then incubated with the appropriate horseradish peroxidase-conjugated secondary antibody. Chemiluminescence was detected and quantified by an ImageQuant LAS 4000 mini system (GE Healthcare Biosci., Pittsburgh, PA, USA) [34, 35].

### Animals

Eight-week-old male BALB/c nude mice were obtained from National Applied Research Laboratories (NARLabs, Taipei, Taiwan) and maintained with a 12 h light/dark cycle and consistent temperature and humidity. All experimental procedures were approved by the Institutional Animal Care and Use Committee of National Chung Hsing University, Taichung, Taiwan (IACUC no. 108-068^R^).

### Orthotopic injection of PDAC tumor cells

PANC1 cells (1 × 10^6^/20 μl) were prepared in a 1:1 cell suspension-Matrigel® mixture. Mice were anesthetized with 2.5% isoflurane. The upper-left abdomen was opened (1 cm long vertically) and the tail of the pancreas was identified below the spleen. The control group (Control) received 20 μl of DPBS-Matrigel® solution and the other two groups, PBS and hAFMSCs, representing the null-treated negative control and stem cell therapy groups, respectively, received 20 μl of 1 × 10^6^ viable tumor cells. All animals received orthotopic injection of either PBS or PANC1 cells on day 0. There were two mice in control group and ten mice in each tumor-bearing mouse with PBS and hAFMSCs transplanted group. Tumor cells were gently injected into the tail of the pancreas and a cotton wool tip was used for compression to prevent leakage. Then, the pancreas was returned back to the abdominal cavity and mice were kept warm until they awakened from anesthesia [36].

### Intravenous injection for cell therapy

Four weeks after orthotopic implantation of PANC1 cells, mice were randomly selected for intravenous injection (1 × 10^6^ cells in 100 μl PBS) of hAFMSCs, which were divided into two groups for single injection treatment (hAFMSCs) or PBS.

### Assessment of primary tumor size

Cardiac terminal blood withdrawal was performed at the time of sacrifice. Pancreases and tumors were resected and the primary tumor size was measured with calipers. The volume was calculated by the following formula: volume (cm^3^) = length (cm) × width (cm) × depth (cm)/2 [37].

### Enzyme-linked immunosorbent assay (ELISA)

Serum was obtained from nude mice on the sacrifice day and a MMP7 ELISA was performed according to the manufacturer’s manual (FineTest, Wuhan, Hubei, China).

### Immunohistochemistry (IHC) analysis

IHC staining was performed as previously described [38]. Briefly, paraffin-embedded tumor sections were incubated with the Ki67 primary antibody (Dako, Carpinteria, CA, USA) at a 1:50 dilution overnight at 4 °C. Sections were incubated with the appropriate biotinylated secondary antibody, stained with 3,3-diaminobenzidine tetrahydrochloride (DAB) and counterstained with hematoxylin the next day.

### Statistics

All data are presented as the mean ± SD. Comparisons between two groups were analyzed using the two-tailed Student’s t-test. For multiple comparisons, one-way ANOVA followed by Tukey’s post hoc test was used to analyze parametric data. All calculations were made using GraphPad Prism software (GraphPad, San Diego, CA, USA). A *P* < 0.05 was considered significant.

## Results

### hAFMSCs exert antiproliferative and antiapoptotic effects on a PDAC-derived cell lines by inducing cell cycle arrest

**Fig. 1:**
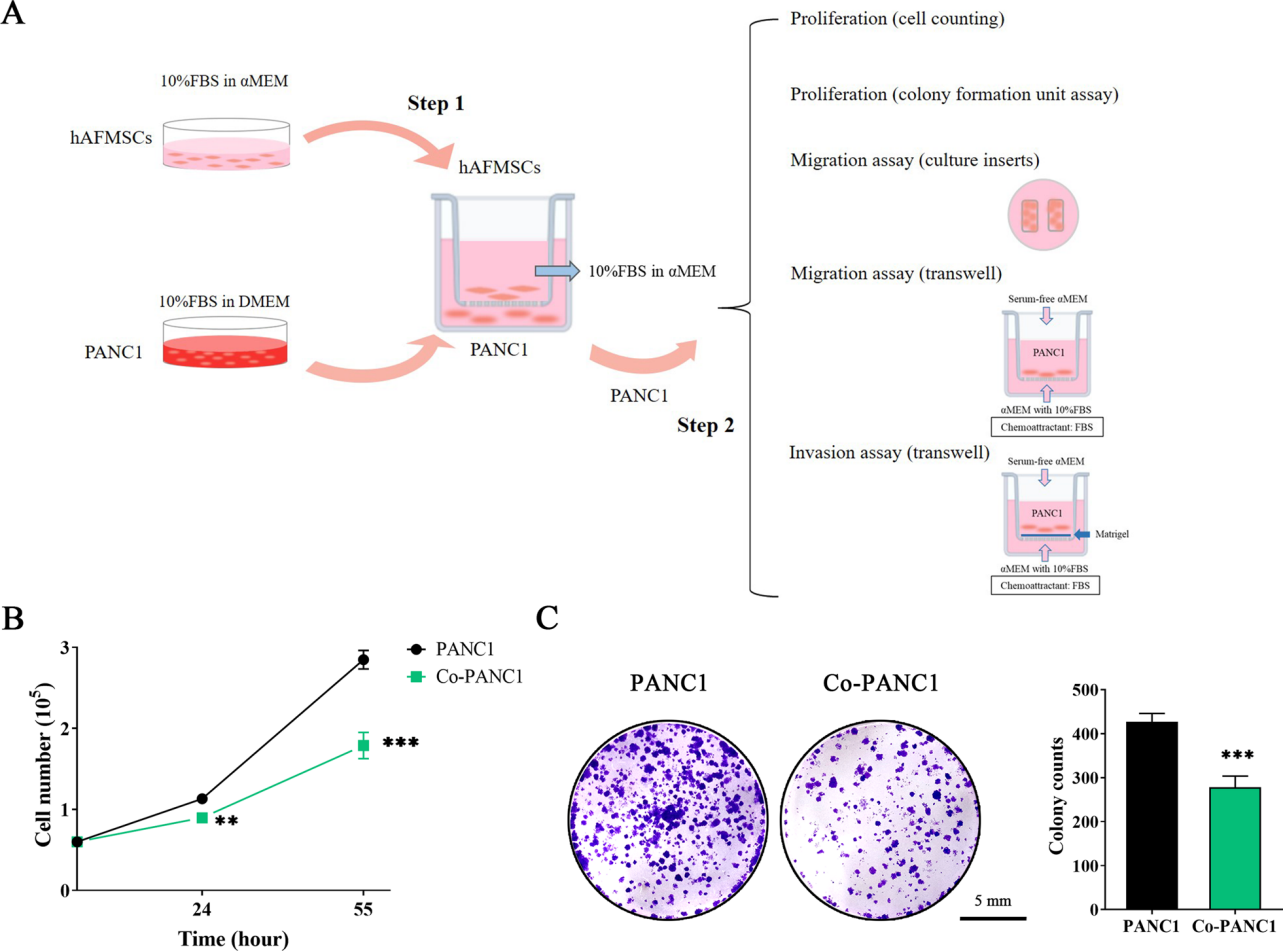
hAFMSCs suppress pancreatic cancer cell proliferation. **A** Schematic for the in vitro hAFMSCs and PANC1 coculture system and functional assay. hAFMSCs and PANC1 cells were placed in the upper Transwell and lower well, respectively (Step 1). After 24 h of incubation, PANC1 cells were harvested from the wells and functional assays were performed, including proliferation assays (cell counting, cell cycle and colony-forming assays), migration assays (2-well culture inserts and Transwell assays) and invasion assays (Step 2). **B** Cell numbers were determined by a hemocytometer at 24 h and 55 h. **C** Representative images of colony formation of crystal violet-stained cells (left panel, scale bar = 5 mm) and quantitative statistics (right panel)

To examine whether hAFMSCs affect cell growth in three pancreatic cancer-derived tumor cell lines, including PANC1, AsPC1 and BxPC3 cells, a cell coculture system was used (Fig. 1A). After 24 h of coculture, PANC1 cells alone and PANC1 cells cocultured with hAFMSCs (Co-PANC1 cells) were incubated in normal culture conditions for 24 h and 55 h. Co-PANC1 cells showed decreased cell numbers by approximately 25% compared to PANC1 cells cultured alone (8.96 ± 0.58 × 10^4^ vs. 11.31 ± 0.53 × 10^4^; *P* < 0.01; Fig. 1B) at 24 h postincubation and by approximately 60% compared to PANC1 cells cultured alone (17.88 ± 1.62 × 10^4^ vs. 28.48 ± 1.15 × 10^4^; *P* < 0.001; Fig. 1B) at 55 h postincubation. As anticipated, similar inhibitory effects on tumor cell growth were shown in AsPC1 and BxPC3 cells (see Additional file 3: Figure S2A). After 24 h of coculture, AsPC1 cells showed decreased cell numbers by approximately 19% compared to AsPC1 cells cultured alone (2.78 ± 0.21 × 10^6^ vs. 3.43 ± 0.31 × 10^6^; *P* < 0.05; Additional file 3: Figure S2A, left); BxPC3 cells showed decreased cell numbers by approximately 20% compared to BxPC3 cells cultured alone (1.21 ± 0.12 × 10^6^ vs. 1.51 ± 0.08 × 10^6^; *P* < 0.05; Additional file 3: Figure S2A, right).

Moreover, a colony-formation assay was performed. After 10 days of incubation, representative images of each condition were acquired (Fig. 1C, left) and the colony numbers were quantified. The results showed that Co-PANC1 cells formed fewer colonies than PANC1 cells alone (278 ± 26 vs. 427 ± 19; *P* < 0.001; Fig. 1C, right). AsPC1 and BxPC3 cell lines showed similar results that much less colonies were formed, an approximately 24% and 12% reduction, respectively, in the coculture conditions than in the tumor cells alone (AsPC1: 185 ± 18 vs. 142 ± 29; BxPC3: 155 ± 17 vs. 138 ± 9; *P* < 0.05; Additional file 3 Figure: S2B). Thus, these results indicated that hAFMSCs effectively suppress cell proliferation activity in three pancreatic cancer-derived tumor cell lines.

The development of fewer colonies might indicate that the effect of hAFMSCs on PANC1 was due to the regulation of replication, resulting in fewer and smaller colonies.

**Fig. 2:**
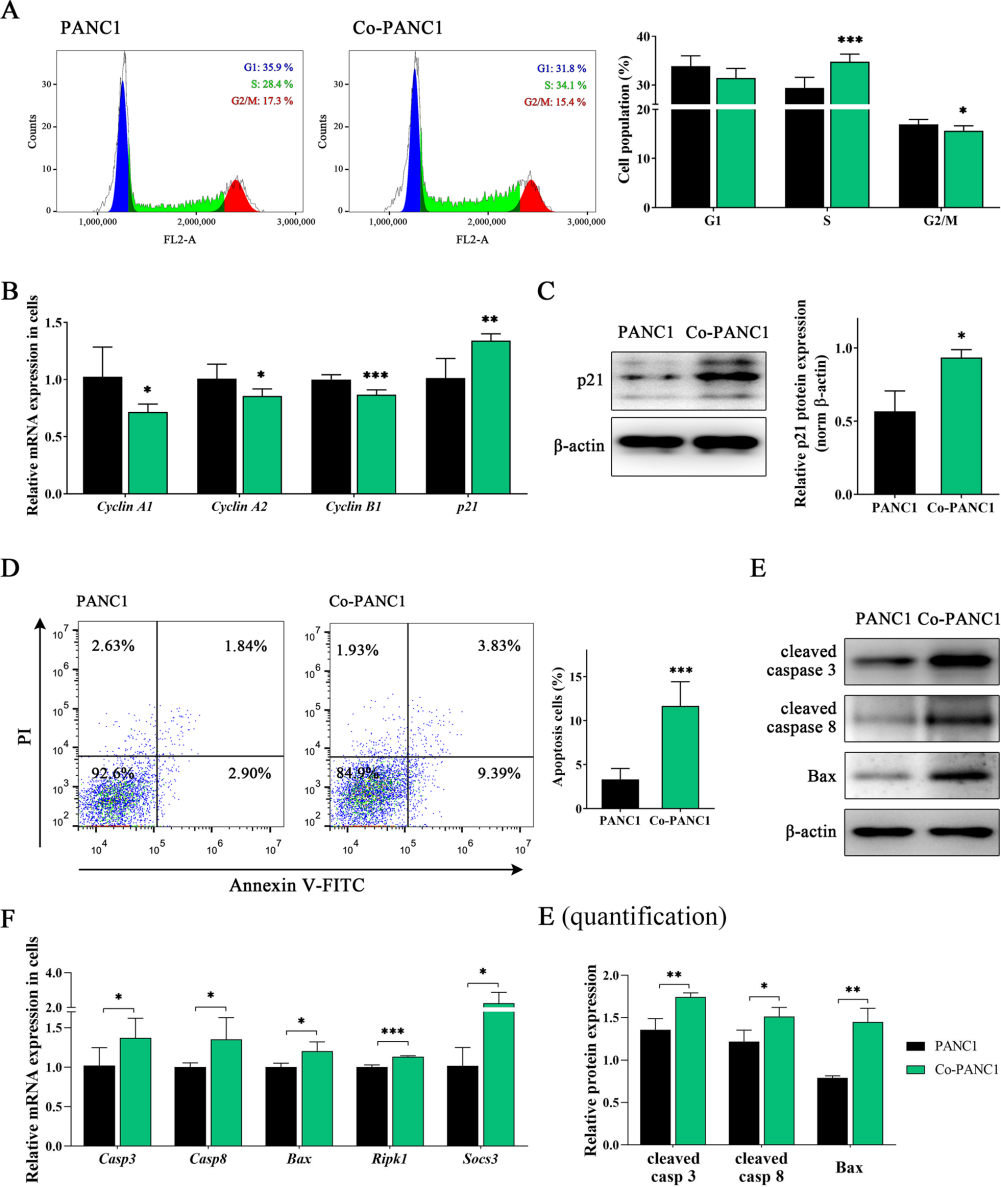
hAFMSCs incude pancreatic cancer cell cycle arrest and apoptosis. **A** Cell cycle analysis of PANC1 cells alone or PANC1 cells cocultured with hAFMSCs for 24 h using flow cytometric analysis (left panel) and the percentages of each cell population at the different cell cycle phases were quantified (right panel). **B** Relative mRNA expression of cell cycle genes in PANC1 and Co-PANC1 cells. **C** (left) Western blot analysis of p21 in the cells and quantification is shown in the right panel. **D** Annexin V/propidium iodide (PI) staining of PANC1 cells alone or PANC1 cells cocultured with hAFMSCs for 48 h using flow cytometric analysis (left panel) and the percentages of apoptotic cell population were quantified (right panel). **E** Western blot analysis of pro-apoptotic molecules, including cleaved caspase3/8 and Bax in the cells and quantification is shown in the bottom panel. **F** Relative mRNA expression of pro-apoptotic genes in PANC1 and Co-PANC1 cells. Data are presented as the mean ± SD of 3 independent experiments. *P < 0.05, **P < 0.01 and ***P < 0.001 versus the PANC1 group by Student’s t-test

Thus, the results of cell proliferation and colony formation assays led us to investigate the role of hAFMSC-induced cell cycle arrest of PANC1 cells (Fig. 2A). The results showed a 6% increase in the proportion of cells in S phase in Co-PANC1 cells compared to PANC1 cells (35.14 ± 1.43 vs. 28.70 ± 1.46; *P* < 0.001; Fig. 1D, right). Correlated with the significant cell cycle arrest in S phase in PANC1 cells treated with hAFMSCs, the expression of cell cycle regulatory genes, including *cyclin A1*, *cyclin A2* and *cyclin B1*, was significantly downregulated, but the level of *p21* was significantly upregulated (Fig. 2B). Western blot results also found that p21 expression level showed about 65% increment in Co-PANC1 cells compared to PANC1 cells (Fig. 2C). The cell viabilities between PANC1 cocultures with and without hAFMSCs at 24 h coculture were also detected. There was no significant difference in the percentage of apoptotic cells in Co-PANC1 cells when compared to PANC1 cells at 24 h coculture (see Additional file 4: Figure S3A), and the mRNA and protein expression of pro-apoptotic related genes, including *Caspase 3/8*, *Bax*, *Ripk1*, and *Socs3*, showed no significant difference between PANC1 and Co-PANC1 (see Additional file 4: Figures S3B and S3C). However, after 48 h coculture with hAFMSCs, the apoptotic cell significantly increased in Co-PANC1 cells compared to PANC1 cells (11.67 ± 2.74 vs. 3.32 ± 1.23; *P* < 0.001; Fig. 2D). qRT-PCR data showed that there was a significant upregulation in the expression levels of the pro-apoptotic-related genes in Co-PANC1 cells (elevated rate is shown as percentage) compared to that in PANC1 cells: 34.5%, *Casp3*; 35.0%, *Casp8*; 20.3%, *Bax*; 13.3%, *Ripk1*; and 120.6%, *Socs3* (*P* < 0.05 or *P* < 0.001; Fig. 2F). Western blot results found that elevated expression level of pro-apoptotic-related molecules in Co-PANC1 cells (elevated rate is shown as percentage) compared to that in PANC1 cells: 28.4%, Casp3; 24.2%, Casp8; and 83.5%, Bax; 83.5% (*P* < 0.05 or *P* < 0.01; Fig. 2E). Thus, these results indicated that hAFMSCs induced pancreatic cancer-derived tumor cell lines cell cycle arrest and apoptosis in a time-dependent manner.

### hAFMSCs inhibit the migration ability of the PDAC-derived cell lines

**Fig. 3:**
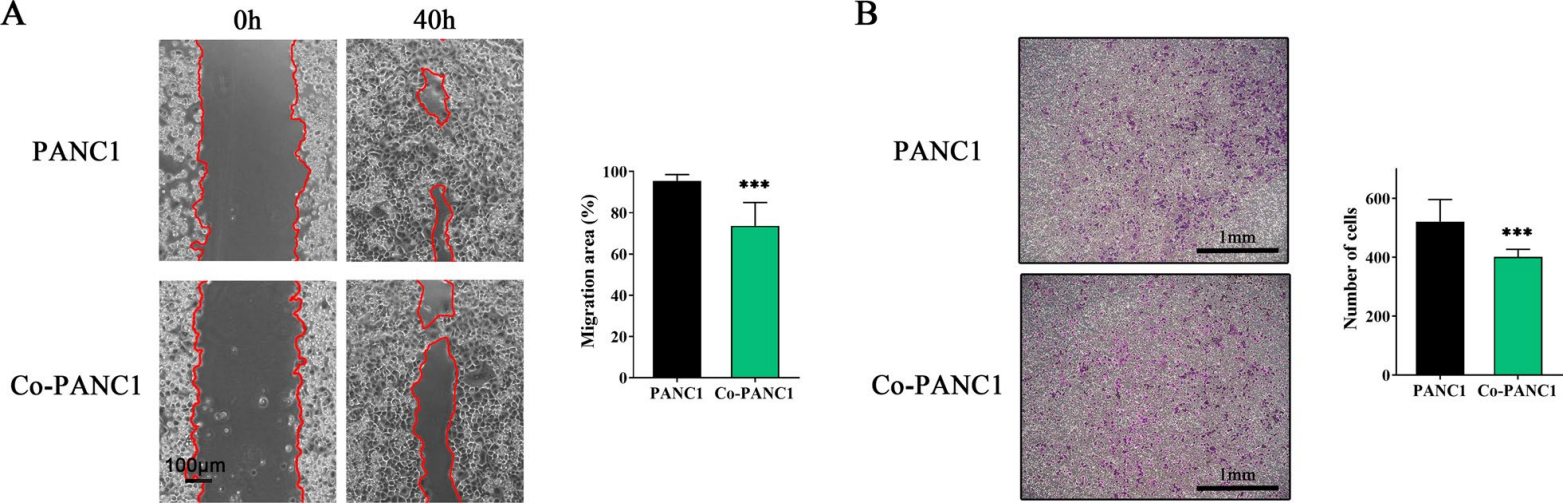
hAFMSCs suppress the migration ability of pancreatic cancer cells. **A** A wound healing assay was used to assess the gap closure rate of PANC1 cells cultured under different conditions at 40 h postincubation. Representative images of gaps at 0 h and 40 h are shown in the left panel and the right panel shows the percentages of cell-free areas at 40 h compared to those at 0 h determined by TScratch (scale bar = 100 μm). **B** Transwell migration assays were performed and the migrated PANC1 cells were stained with crystal violet and counted by ImageJ (scale bar = 1 mm). The quantified data are presented in the right panel. The results are shown as the mean ± SD of 3 independent experiments. ***P < 0.001 versus the PANC1 group by Student’s t-test

To evaluate the migration inhibitory effects of hAFMSCs on PANC1 cells, a wound-healing assay was performed. Representative images showed a similar gap in each group after removal of the 2-well culture insert (Fig. 3A, left). At 40 h postincubation, nearly 85% of the control group (PANC1 cells alone) migrated across the gap area, while merely 74% of the Co-PANC1 cells migrated across the gap area (Fig. 3A, right). Furthermore, Boyden chamber assays showed that the migrating cells in the control group (PANC1 alone) were easily identified (521 ± 75) and significantly fewer transmembrane cells were observed in the Co-PANC1 group (393 ± 23; *P* < 0.001; Fig. 3B). As anticipated, abundant migrating AsPC1 and BxPC3 cells were easily identified on the transmembrane (AsPC1: 127.0 ± 23.3; BxPC3: 279.2 ± 89.4) and fewer transmembrane cells were observed in the Co-AsPC1 and Co-BxPC3 group (Co-AsPC1: 54.0 ± 15.9, *P* < 0.01; Co-BxPC3: 138.8 ± 53.2, *P* < 0.05; Additional file 3: Figure S2C). Thus, these results indicated that hAFMSCs suppress pancreatic cancer-derived tumor cell lines migration.

### hAFMSCs inhibit the invasion ability of PDAC-derived cell lines

**Fig. 4:**
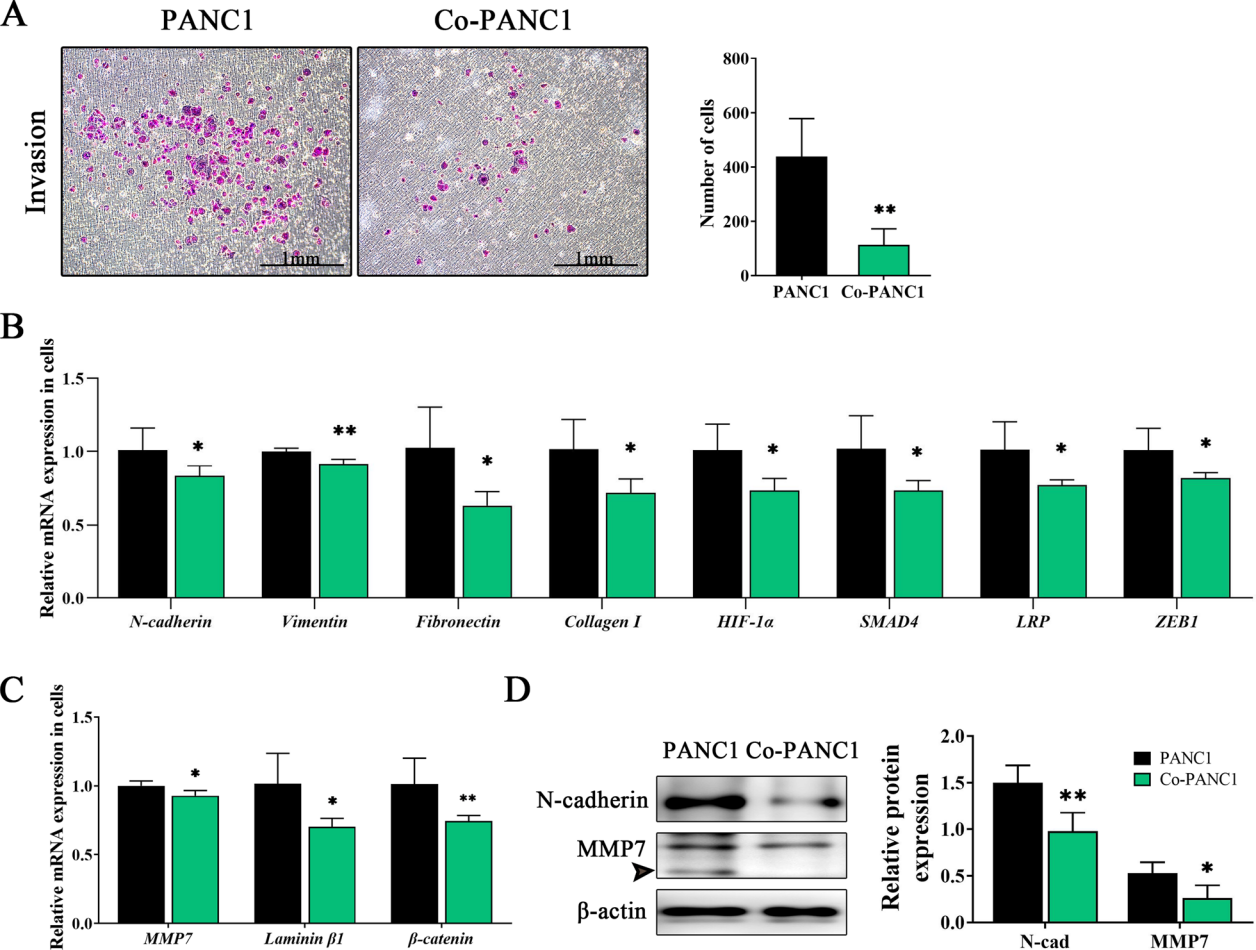
hAFMSCs suppress the invasive ability of pancreatic cancer cells through MMP7-mediated EMT. **A** The invasive capacity was determined by a Boyden chamber-Matrigel assay. The representative images are shown on the left are migrated cells stained with crystal violet and counted by ImageJ and the quantified data are presented in the right panel (scale bar = 1 mm). qRT-PCR was per-formed to measure the mRNA expression of **B** EMT-related genes and **C** MMP7-related genes between the PANC1 alone and Co-PANC1 groups. **D** (left) Western blot analysis of N-cadherin and MMP7 in the cells and quantification is shown in the right panel. The results are shown as the mean ± SD of 3 independent experiments. *P < 0.05 and **P < 0.01 versus the PANC1 group by Student’s t-test

PANC1 cells with or without coculture of hAFMSCs were seeded in Transwell inserts precoated with Matrigel and after incubation for 48 h, the migrating cells trapped in the membrane were quantified. Representative images showed that more PANC1 cells invaded through the membrane (Fig. 4A, left) than Co-PANC1 cells (438.67 ± 139.55 vs. 113.67 ± 58.4; *P* < 0.01; Fig. 4A, right). Epithelial-mesenchymal transition (EMT) acts as a key regulator of the invasion process. The mRNA levels of genes that contribute to EMT targets, including N-cadherin, vimentin, fibronectin and collagen I, as well as EMT-signaling molecules, including HIF-1α, SMAD4, LRP and ZEB1, were detected. qRT-PCR data showed that there was a significant downregulation in the expression levels of the following EMT-signaling pathway molecules and downstream targets in Co-PANC1 cells (reduction rate are shown as percentage) compared to that in PANC1 cells: 18.2%, *N-cadherin*; 8.5%, *vimentin*; 40.9%, *fibronectin*; 30.7%, *collagen I*; 28.3%, *HIF-1α*; 29.3%, *SMAD4*; 25.2%, *LRP* and 19.5%, *ZEB-1* (*P* < 0.05 or *P* < 0.01; Fig. 4B). Western blot results found that N-cadherin expression level showed about 35% decrement in Co-PANC1 cells when compared to PANC1 cells (Fig. 4D).

MMP7 plays a crucial role in the tumor cell invasion cascade and migration through the activation of β-catenin signaling. Significant inhibition of EMT signaling associated with invasion and migration ability in Co-PANC1 cells was observed. The mRNA expression levels of *MMP7* and its downstream signaling molecules were then detected. The mRNA level of *MMP7* showed a 10% reduction in Co-PANC1 cells compared to PANC1 cells (*P* < 0.05; Fig. 4C). Furthermore, there was a significant reduction in the expression of the downstream target genes of MMP7 in Co-PANC1 cells with an approximate 28% reduction in β-catenin and a 32% reduction in laminin β1 compared to that in PANC1 cells (Fig. 4C). Western blot results found that MMP7 expression level showed about 50% increment in Co-PANC1 cells when compared to PANC1 cells (Fig. 4D). These results indicated that PANC1 cells treated with hAFMSCs have reduced invasion and migration abilities partly through inhibition of MMP7 signaling-triggered EMT.

### Transplantation of hAFMSCs suppresses the growth of PANC1 tumors in orthotopic xenograft mice

**Fig. 5:**
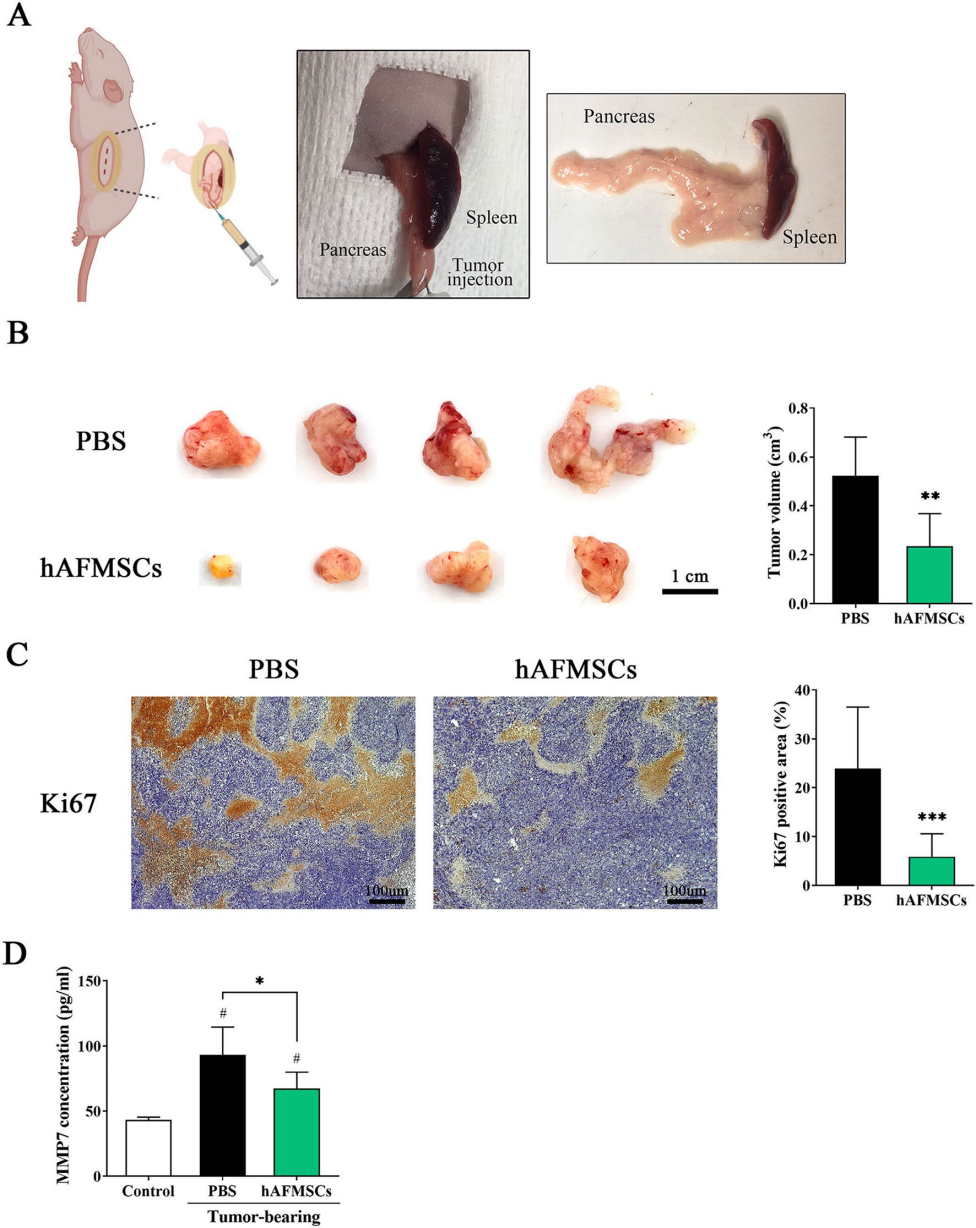
Transplantation of hAFMSCs suppresses the growth of tumors in an orthotopic xenograft mouse model. **A** Schematic procedure of incision location and orthotopic injection of pancreatic cancer cells (left panel). The macroscopic appearance of pulp filled with orthotopic tumors within the pancreatic tail (middle panel) and after excision (right panel), where the small module indicates the injection site. Four weeks after orthotopic injections of PANC-1 cells, mice randomly received PBS (PBS group) or hAFMSCs (hAFMSC group). **B** (left panel) Eight weeks after hAFMSCs transplantation, tumors disassociated from the pancreas (scale bar = 1 cm), **B** (right panel) tumor volumes were quantified and measured and tumor volume was calculated using the following formula: 1/2 × length × width × depth. **C** Immunohistochemistry staining for Ki67 in the pancreatic tumor site. Scale bar = 100 μm. The area of the Ki67-positive signal (brown) was calculated in 3 fields from three independent experiments and it is represented as the percentage (%) of the positive area. The results are shown as the mean ± SD of 3 independent experiments. **P < 0.01 and ***P < 0.001 versus the PBS group by Student’s t-test. **D** Serum MMP7 levels were detected by ELISA 3 months after tumor cell implantation. Control indicates mice without orthotopic xenograft surgery. The results are shown as the mean ± SD of 3 independent experiments. One-way ANOVA was used. *P < 0.05 versus PBS, ^#^*P* < 0.05 versus control

We next investigated the inhibitory effects of hAFMSCs on PANC1 cell proliferation, migration and invasion activities to reduce PDAC tumors in vivo. Thus, a tumor-bearing mouse model was established in nude mice following orthotopic xenografting of PANC1 cells. Four weeks following PANC1 cell implantation, a single dose of hAFMSCs (10^6^ cells) or PBS was intravenously injected into nude mice (Fig. 5A). Three months after tumor cell implantation, tumors were disassociated from the pancreas (Fig. 5B, left). Tumors of PBS treated tumor-bearing mice (PBS) grew to 0.52 ± 0.16 cm^3^ in size, but the size of PANC1 tumors on mice that received hAFMSCs injection (hAFMSCs) was significantly reduced (0.23 ± 0.13 cm^3^; *P* < 0.01; Fig. 5B, right). In addition, Ki67 immunohistochemistry (IHC) staining was used to measure tumor cell proliferation. The Ki67-positive cells were significantly lower in the hAFMSC injection group than in the PBS-treated group (5.88 ± 4.68% vs. 23.88 ± 12.58%; *P* < 0.001; Fig. 5C), which was consistent with the decreased size (Fig. 5B).

Mice without tumor cell implantation had serum MMP7 levels below 43.33 ± 2.10 pg/ml (control), whereas at 3 months following PANC1 tumor cell implantation, MMP7 levels were significantly higher (93.29 ± 12.62 pg/ml; PANC1 alone group). However, the serum MMP7 levels in the PANC1 tumor-bearing mice that received hAFMSCs transplantation were significantly reduced (67.38 ± 21.28 pg/ml; hAFMSCs; Fig. 5D).

### Transplantation of hAFMSCs inhibits PANC1 tumor metastasis in orthotopic xenograft mice

**Fig. 6:**
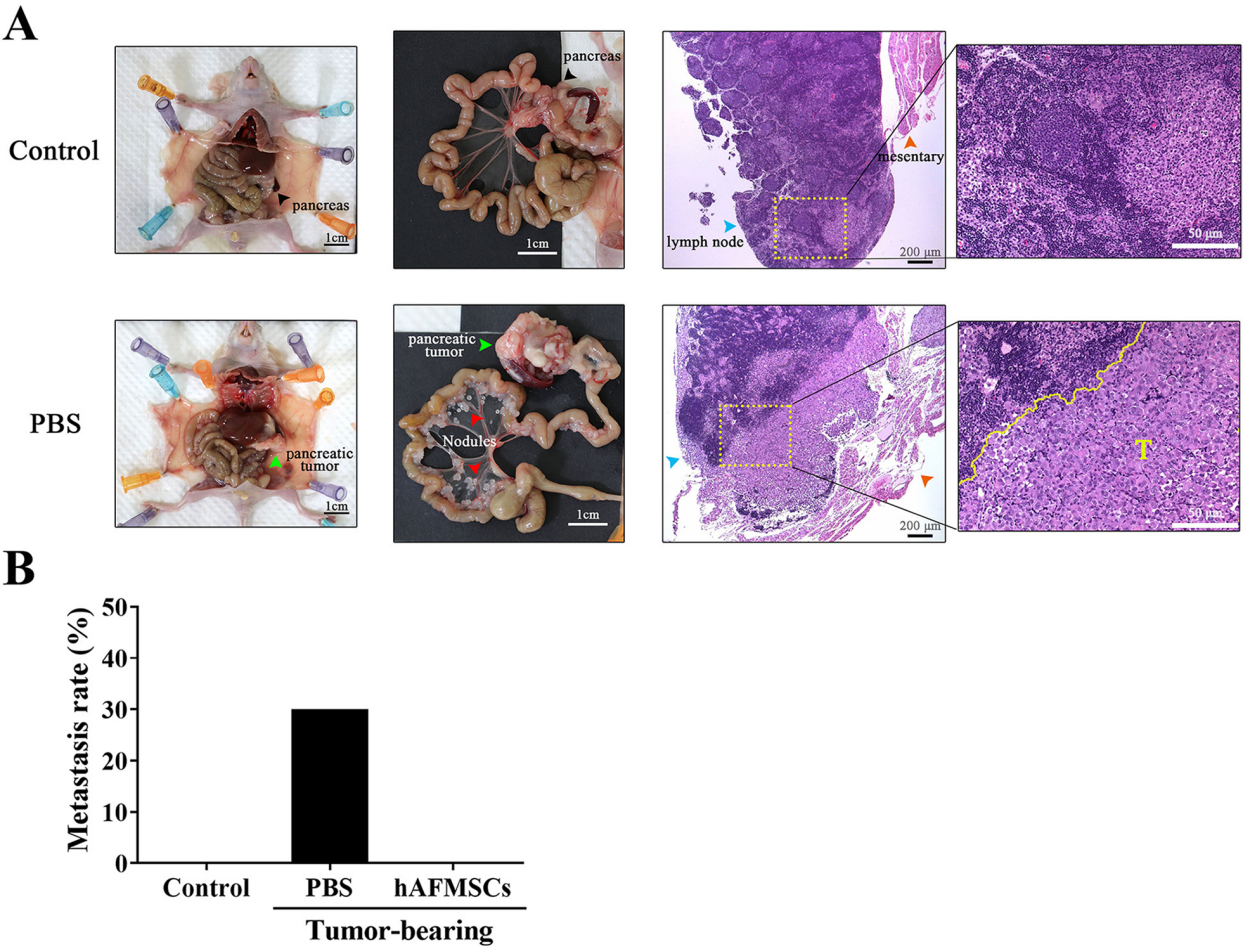
hAFMSCs transplantation suppresses metastatic nodules in an orthotopic xenograft mouse model. **A** (left) Representative images demonstrating the whole-body dissection (ventral view) of nude mice receiving PBS orthotopic injection (control) and pancreatic cancer cells (PANC1). Green arrowhead indicates orthotopic pancreatic tumors. Scale bar = 1 cm. **A** (middle) Representative images of aligned intestines of control and PANC1 mice are shown and numerous nodules were observed on the mesentery of mice receiving PANC1 cell orthotopic injection. Red arrowhead indicates metastatic tumor nodules. Scale bar = 1 cm. **A** (right) Representative H&E staining images of metastatic nodules surround mesenteric lymph nodes are shown. Scale bar = 200 μm. Blue arrowhead indicates mesenteric lymph nodes and orange arrowhead indicates mesentery tissue. The magnified images were area within yellow dotted line and presented next to the H&E staining images. T: tumor cells. Scale bar = 50 μm. **B** Metastasis rate of nude mice under different treatments as follows: Control group, PANC1 implantations with PBS (PBS group) and with hAFMSCs intravenous injection (hAFMSCs group)

Orthotopic xenografts of PANC1 tumors in nude mice represent a known attractive metastatic model (Fig. 6A, left). Secondary organ metastasis, on mesentery (Fig. 6A, middle), was observed with the discovery of cancer cells surrounding mesenteric lymph node under H&E stained sections examination (Fig. 6A, right). From which, the mesentery metastatic nodules might be resulted from the migration of orthotopic PANC1 cancer cells in pancreas and eliminated the possibility from the leakage of cancer cells implantation. As predicted from the in vitro PANC1 invasion ability data (Fig. 4), the rate of secondary organ metastasis was significantly less in hAFMSC-treated mice compared to that in the PBS-treated group. In fact, peritoneal dissemination (30%) were observed in nude mice after PANC1 orthotopic xenografts were established (Fig. 6B), but no metastasis was detected in hAFMSC-treated group. In addition, three mice with metastatic tumors showed higher serum MMP7 levels (112.13 ± 22.30 pg/ml; *n* = 3) than the nonmetastatic PANC1 tumor-bearing mice (81.98 ± 10.87 pg/ml; *n* = 7; *P* < 0.05).

### Molecular mechanisms of engrafted hAFMSCs on tumor growth inhibition in an orthotopic xenograft mouse model

**Fig. 7:**
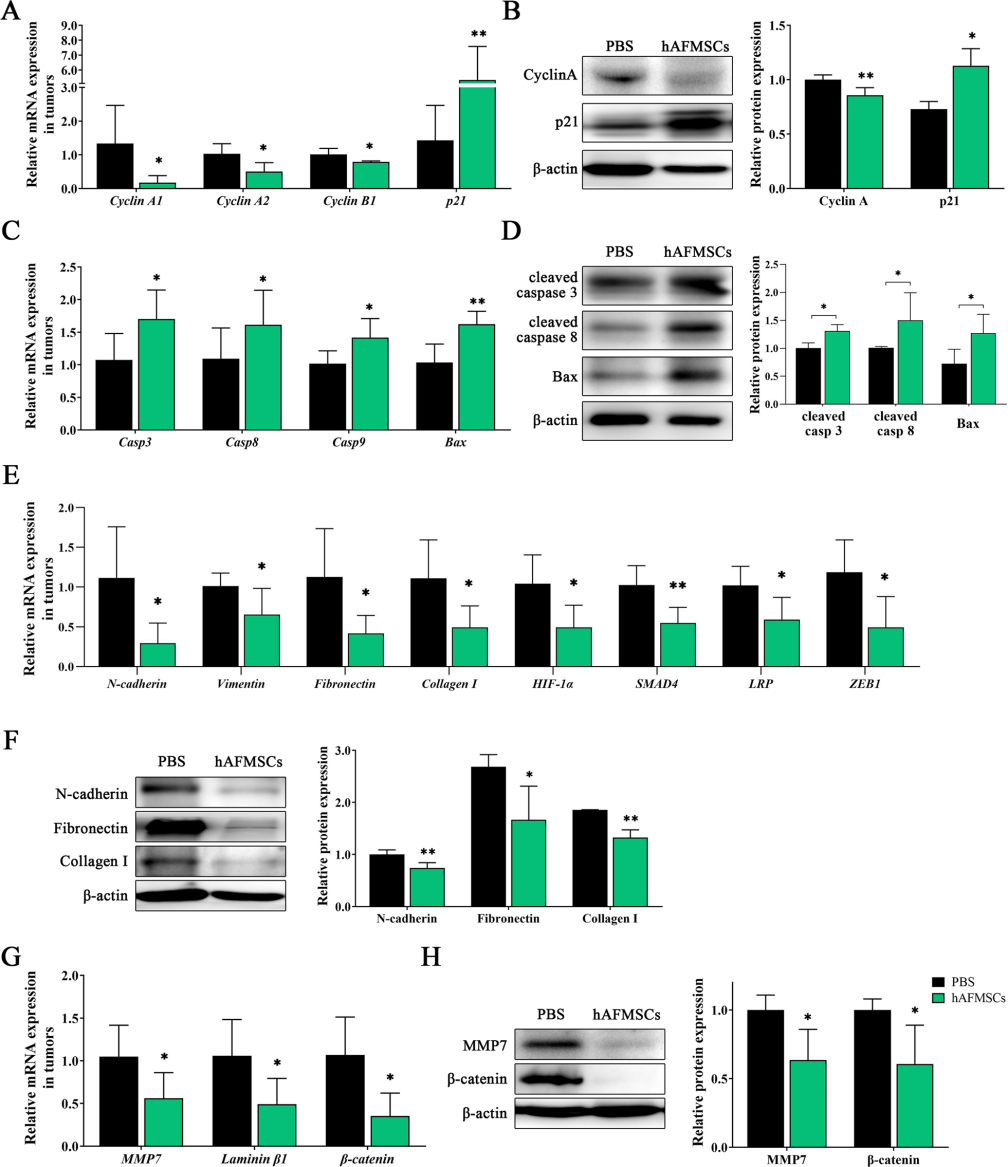
Mechanisms of tumor growth inhibition by engrafted hAFMSCs in an orthotopic xenograft mouse model. **A**, **C**, **E**, **G** Quantitative RT-PCR and **B**, **D**, **F**, **H** western blot analysis was performed to analyze the expression of **A**, **B** cell cycle-, **C**, **D** pro-apoptotic-, **E**, **F** EMT-, and **G**, **H** MMP7-related genes in the PANC1 tumors of orthotopic xenograft mice that received hAFMSCs or PBS. Values were normalized to the β-actin gene and are expressed relative to the PANC1 group. Human β-actin was used as a normalization control. The results are shown as the mean ± SD of 3 independent experiments. *P < 0.05 and **P < 0.01 versus the PANC1 group by Student’s t-test

To determine the inhibitory effects of hAFMSCs on the proliferation and MMP7 signaling-triggered EMT in PANC1 tumor-bearing mice, the expression levels of cell cycle- and EMT-related mediators were determined. The mRNA levels of cell cycle regulatory genes, including *cyclin A1* (120% reduction), *cyclin A2* (54% reduction) and *cyclin B1* (23% reduction), were significantly downregulated, but the mRNA level of *p21* (400% increment) was significantly upregulated in the hAFMSCs injection group compared to that in the PBS-treated group (Fig. 7A). Western blot results also found that 14.16% reduction in Cyclin A and 54.4% increment in p21 in the PANC1 tumor-bearing mice that received hAFMSCs injection compared to PBS-treated PANC1 tumor-bearing mice (Fig. 7B and also see Additional file 5: Figure S4). The mRNA levels of pro-apoptotic-related genes, including *Casp3* (58.7% increment), *Casp8* (47.7% increment), *Casp9* (39.5% increment) and *Bax* (56.8% increment), were significantly upregulated in the hAFMSCs injection group compared to that in the PBS-treated group (*P* < 0.05 and *P* < 0.01; Fig. 7C). Western blot results also found that 30.2% increment in cleaved caspase3, 48.7% increment in cleaved caspase8, and 76.0% increment in Bax in the PANC1 tumor-bearing mice that received hAFMSCs injection compared to PBS-treated PANC1 tumor-bearing mice (*P* < 0.05; Fig. 7D). Furthermore, the expression levels of various factors mediating EMT were significantly downregulated in the hAFMSCs injection group compared to those in the PBS group as follows: 84.6% reduction in *N-cadherin*, 36.0% reduction in *vimentin*, 75.3% reduction in *fibronectin*, 66.3% reduction in *collagen I*, 56.6% reduction in *HIF-1α*, 55.4% reduction in *SMAD4*, 43.9% reduction in *LRP* and 76.9% reduction in *ZEB-1* (*P* < 0.05 and* P* < 0.01; Fig. 7E). Western blot results also found that 25.87% reduction in N-cadherin, 37.85% reduction in Fibronectin, and 28.73% reduction in Collagen I in the PANC1 tumor-bearing mice that received hAFMSCs injection compared to PBS-treated PANC1 tumor-bearing mice (*P* < 0.05 and* P* < 0.01; Fig. 7F and also see Additional file 5: Figure S4).In addition, the *MMP7* mRNA level showed a 51.4% reduction in the hAFMSCs injection group and its downstream target genes, *β-catenin* and *laminin β1*, also showed a 73.6% and 59.4% reduction, respectively, in the hAFMSCs injection group compared to the PBS group (*P* < 0.05; Fig. 7G). Moreover, Western blot analyses also showed a 36.56% reduction in MMP7, and 39.45% reduction in β-catenin in the PANC1 tumor-bearing mice that received hAFMSCs injection compared to PBS-treated PANC1 tumor-bearing mice (*P* < 0.05; Fig. 7H and also see Additional file 5: Figure S4).

## Discussion

Pancreatic cancer mouse models have been established using a variety of methods, including a transgenic mouse model with the most representative tumor suppressor gene in PDAC, *Kras*, being mutated or chemically induced in pancreatic cancer [39] and a xenograft mouse model consisting of heterotopic or orthotopic xenografts [40]. The orthotopic PDAC model is the most frequently used and has been well established [41], providing a more practical situation by injecting tumor cells or transplanting tumor mass directly to the pancreas [42] and this model has the advantages of easy replication, high reproductivity and restrained tumor location. According to a previous report [43] that has compared pancreatic tumorigenesis between subcutaneously and orthotopically implanting human pancreatic adenocarcinoma cell lines in mice, primary tumors are obtained at a 100% reproducibility rate in mice with orthotopic injection (8/8 mice) but only a 60% reproducibility rate in mice with subcutaneous injection (3/5 mice). In our study, orthotopic xenografts were used to establish a pancreatic adenocarcinoma mouse model in terms of the advantages of restraining location and its high reproducibility rate with 93% of mice carrying primary tumors.

Despite the extensive use of MSCs in preclinical models and clinical trials, the identity and role of MSCs in tissue repair and organogenesis remain ambiguous [44]. In addition, the type of cross-talk between MSCs and cancer cell both directly and indirectly also exerted no clear conclusion regarding whether the outcome is positive or negative for tumor progression. Doi et al. [45] and Kidd et al. [46] directly inject the MSCs to tumor site showed remarkably attenuate the growth of pancreatic carcinoma cells. Another study demonstrated that human ADMSCs effectively suppress proliferation in vitro and that conditioned medium of ADMSCs also inhibits tumor cell proliferation and induces tumor cell death, therefore decreasing the viability of tumor cells. Thus, ADMSCs might inhibit tumor proliferation ability by influencing the cell cycle. Subcutaneous injection of Capan-1 pancreatic cancer cells in vivo treated with single dose of intratumoral injection of ADMSCs resulted in the volume of tumors being significantly decreased in mice [47]. However, Kabashima-Niibe et al. [48] and Saito et al. [49] reported negative outcomes for human MSCs directly interact with PDAC, as they promote the level of EMT and facilitate cancer progression in PDAC both in vitro and in vivo. On the other hand, MSCs are widely used as cellular vehicles to deliver genes or agents that affect tumor progression through indirect interaction mechanism. Adipose-derived mesenchymal stem cells (ADMSCs) used in cell therapy are also controversial. Coculturing ADMSCs with different pancreatic cancer cell lines results in both the promotion of proliferation and invasion in vitro, possibly through secreting SDF-1; however, xenograft mouse models do not demonstrate these same results. For example, ADMSCs increased tumor volume in a SW1990 xenograft mouse model but did not increase PANC1 xenograft tumor sizes [50]. Many studies have reported that MSCs can be designed as vehicles to deliver agents, such as TRAIL or suicide gene thymidine kinase (TK), or miRNA, such as miR-1231 or miR-126-3p for reducing the pancreatic tumor growth [51]. Taken together, our in vitro transwell coculture experiment confirmed that hAFMSCs attenuated PANC1 cell proliferation through paracrine effects, but the impact of direct MSC-cancer cell interactions should not be ignored, which would be further studied.

Human AFMSCs have unique features of low immunogenicity, low major histocompatibility complex antigen expression level and low inflammation and they incur no ethical objection and are easily available with less restricted differential potential [52]. Thus, hAFMSCs have been used to treat various diseases and cancers. In this study, our data indicated that either a single injection or multiple intravenous injections of hAFMSCs result in similar efficient homing of the cells to the tumor site (see Additional file 6: Figure S5A) and decreased tumor volume (see Additional file 6: Figure S5B) may through attenuation of cell proliferation and EMT, in which malignant cancer cells migrate to the next target. For the purpose of acquiring more nutrients, invasion of tissues followed by the epithelial-mesenchymal transition process is necessary in an orthotopic xenograft animal model [53].

MMP7 may act as a key driver of PDAC initiation, progression, invasion and metastasis [54]. Chatterjee et al. mentioned that MMP7 contribute to an upstream signal for EMT responses and also degrade the peritumoral stroma, which may facilitate PDAC tumor cell EMT, invasion and metastasis; thus, MMP7 has been regarded as a metastasis indicator when the concentration is increased [55, 56]. Taking advantage of MMP7 deficient mouse models, the EMT-related transcription factors, including Snail, Slug, Twist, Smad4 and ZEB1 [57], and the several ECM components, including Fibronectin, Collagen, Vimentin and N-cadherin, were reduced in tumor progression and fibrotic disease model [55, 58]. A previous study has also reported that a high serum MMP7 expression level is considered a useful clinical candidate to predict tumor stage and survival in patients suffering from pancreatic ductal adenocarcinoma [59] and other types of cancers, including colon, ovarian and digestive system cancers [60]. Fang et al. found that chemoendocrine therapy can inhibit colon tumor cell invasion, migration and cell proliferation via the downregulation of MMP7 [61]. Furthermore, Zhang et al. used an RNAi-mediated silencing tool to decrease MMP7 levels to not only attenuate colon tumor cell proliferation but also elevate cancer cell radiotherapy or chemotherapy sensitivity [62]. Guo et al. found that miR-508-3p directly downregulates MMP7 to inhibit ovarian cancer cell proliferation, migration and invasion [63]. In addition, molecules, including uroplakin 1A (UPK1A) [64] and RBMS3 [65], have been proposed to possess the ability to induce cell cycle arrest at G1/S phase and inhibit tumor invasion and metastasis, which may be mediated by the downregulation of MMP7 in esophageal squamous cell carcinoma or nasopharyngeal carcinoma. Thus, MMP7 may act as a promising therapeutic candidate for PDAC treatment.

## Conclusion

In this study, the positive effects of hAFMSCs on PANC1 cell proliferation, migration and invasion were observed in in vitro cell cultures and verified in a PANC1 orthotopic xenograft mouse model. hAFMSCs may be able to modulate the cell cycle and EMT of PANC1 cells, possibly through the MMP7 signaling pathway. Thus, hAFMSCs may act as a promising therapeutic candidate for clinical PDAC treatment.

## Supplementary Information

**Additional file 1. Figure S1: **Characterization of human amniotic fluid mesenchymal stem cells (hAFMSCs) obtained from Dr. Huang’s lab. Immunophenotypes of hAFMSCs by flow cytometric analysis, and all samples were prepared according to the manufacturer’s manual of the BD Stemflow Human MSC Analysis Kit. (A-C) Cells stained with isotype control antibody and were detected in the FL2, FL3, and FL4channels. Cells were positive for cell surface antigens (D) CD73, (E) CD44, and (F) CD105, but negative for CD11b, CD19, CD34, CD45, HLA-DR.

**Additional file 2. Table S1: ** Oligonucleotide primers used for qRT-PCR analysis in this study.

**Additional file 3. Figure S2: ** hAFMSCs suppress cell proliferation in pancreatic cancer cell lines, AsCP1 and BxPC3. (A) Cell numbers of AsPC1 (left) and BxPC3 (middle) were determined by a hemocytometer at 24 h post coculture and quantified (right). (B) Representative images of colony formation of crystal violet-stained cells (left panel) and quantitative statistics (right panel). (C) Represented images were migrated cells stained with crystal violet (left panel) and counted by ImageJ. The quantified data are presented in the right panel. The results are shown as the mean ± SD of 3 independent experiments. * P < 0.05 and ** P < 0.01 vs. the Control group by Student’s t-test.

**Additional file 4. Figure S3: ** Cytotoxicity of PANC1 cells cocultured with hAFMSCs. (A) Cells treated with 500 μM hydrogen peroxide for 1 hours, and then stained with annexin V-FITC only. This group was used for setting up the flow cytometric compensation and the set the quadrant gates to separate four populations. After 24 h of coculture, PANC1 and Co-PANC1 cells were assessed by flow cytometric analysis with Annexin V/propidium iodide (PI) staining. The percentages of Annexin V-positive cells (apoptotic cell population) are shown. (B) Relative mRNA expression of pro-apoptotic genes in PANC1 and Co-PANC1 cells. (C) Western blot analysis of pro-apoptotic molecules, including cleaved caspase3/8 and Bax in the cells and quantification is shown in the right panel. Data are presented as the mean ± SD of 3 independent experiments. ns. P > 0.05 vs. the PANC1 group by Student’s t-test. The data are presented as the mean ± SD of 3 independent experiments with 3 technical replicates.

**Additional file 5. Figure S4: ** Original images of Western blots. The original Western blot images for p21 and β-actin are shown in Figure 2C; for cleaved caspase3, cleaved caspase8, Bax, and β-actin are shown in Figure 2E; for N-cadherin, MMP7, and β-actin are shown in Figure 4D. Original images of Western blots. The original Western blot images for CyclinA, p21, and β-actin are shown in Figure 7B; for cleaved caspase3, cleaved caspase8, Bax, and β-actin are shown in Figure 7D; for N-cadherin, fibronectin, collagen I, and β-actin are shown in Figure 7F; for MMP7, β-catenin, and β-actin are shown in Figure 7H.

**Additional file 6. Figure S4 (cont.): ** Original images of Western blots. The original Western blot images for CyclinA, p21, and β-actin are shown in Figure 7B; for cleaved caspase3, cleaved caspase8, Bax, and β-actin are shown in Figure 7D; for N-cadherin, fibronectin, collagen I, and β-actin are shown in Figure 7F; for MMP7, β-catenin, and β-actin are shown in Figure 7H.

**Additional file 7. Figure S5: ** In vivo CM-Dil-labeled hAFMSC tracing. (A) After four weeks of orthotopic PANC1 cancer cells implantation, mice were randomly divided into single or multiple (once a week) intravenous injection of CM-DiI-stained hAFMSCs (1 × 106 cells in 100 μl PBS/mouse). The recipient mice were maintained for 4 weeks after their first dose of intravenous injection, and then tumors were disassociated from the pancreas. DAPI staining of nuclei (blue). Migrated hAFMSCs located in tumor sites (red). Scale bar = 100 μm. (B upper) Representative images are tumors disassociated from pancreas and aligned with the scale bar of 1 cm. (B bottom) Tumor volumes were calculated with the formula: 1/2 x length x width x depth. Data are demonstrated in mean ± SD, one-way ANOVA followed by Tukey’s post hoc test were performed in comparison with PBS group, *P <0.05.

## Data Availability

All data generated or analyzed during this study are included in this published article and its Additional files.

## Abbreviations

PDAC: Pancreatic ductal adenocarcinoma
hAFMSCs: Human amniotic fluid mesenchymal stem cells
EMT: Epithelial-mesenchymal transition
MMP7: Matrix metallopeptidase 7
BM-MSCs: Bone marrow-derived mesenchymal stem cells
α-MEM: Minimum essential medium α
FBS: Fetal bovine serum
bFGF: Bovine fibroblast growth factor-basic
PBS: Phosphate-buffered saline
PI: Propidium iodide
qRT-PCR: Quantitative real-time polymerase chain reaction
ELISA: Enzyme-linked immunosorbent assay
IHC: Immunohistochemistry
ADMSCs: Adipose-derived mesenchymal stem cells
