# Supplementary figures and images for: Human amniotic fluid mesenchymal stem cells attenuate pancreatic cancer cell proliferation and tumor growth in an orthotopic xenograft mouse model
Source: Stem Cell Res Ther. 2022 Jun 3;13:235. doi: 10.1186/s13287-022-02910-3 (PMC9166578; doi:10.1186/s13287-022-02910-3)

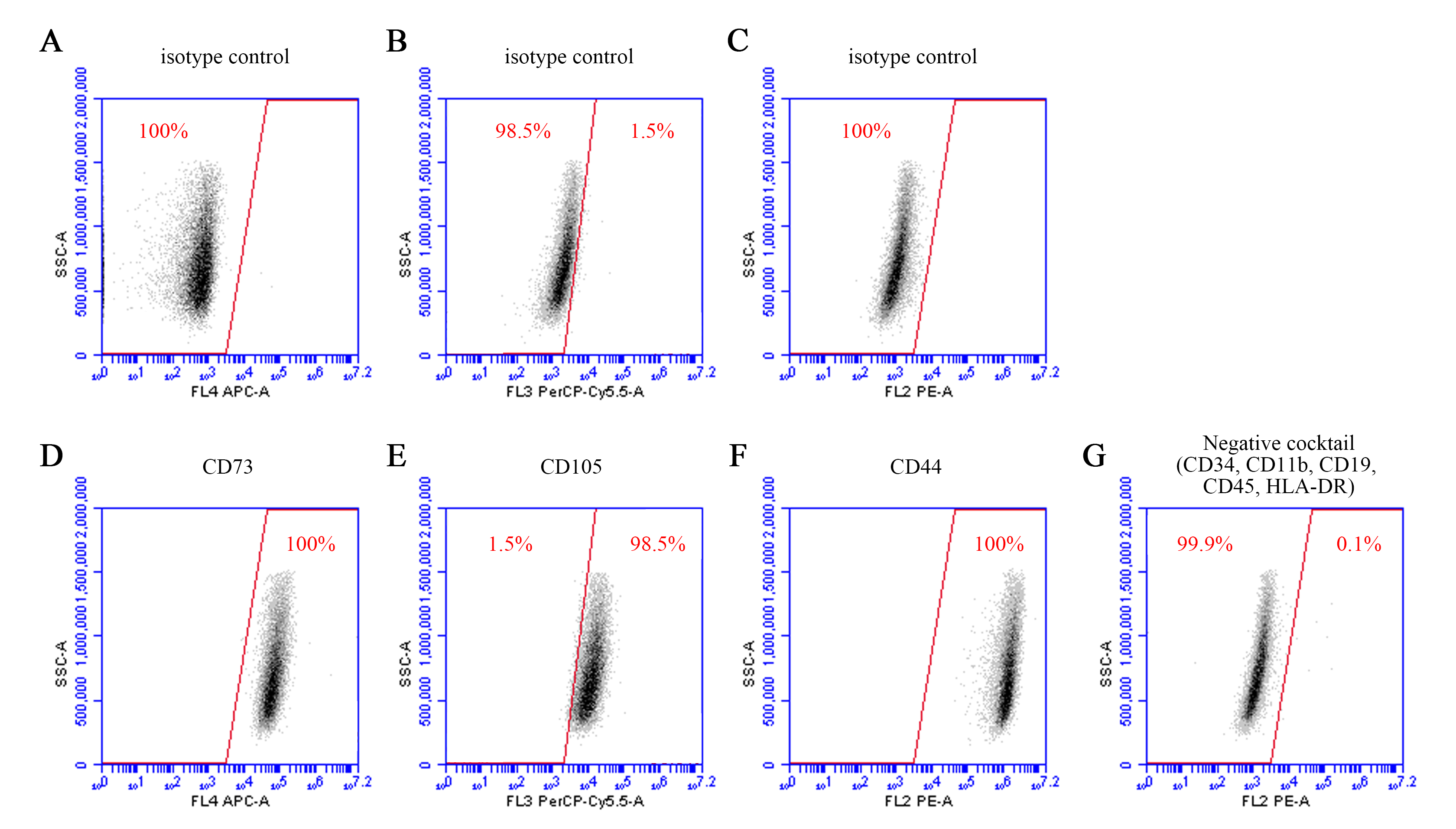

Supplement: Supplementary file 1 — Additional file 1. Figure S1: Characterization of human amniotic fluid mesenchymal stem cells (hAFMSCs) obtained from Dr. Huang’s lab. Immunophenotypes of hAFMSCs by flow cytometric analysis, and all samples were prepared according to the manufacturer’s manual of the BD Stemflow Human MSC Analysis Kit. (A-C) Cells stained with isotype control antibody and were detected in the FL2, FL3, and FL4channels. Cells were positive for cell surface antigens (D) CD73, (E) CD44, and (F) CD105, but negative for CD11b, CD19, CD34, CD45, HLA-DR. [file 13287_2022_2910_MOESM1_ESM.tif]

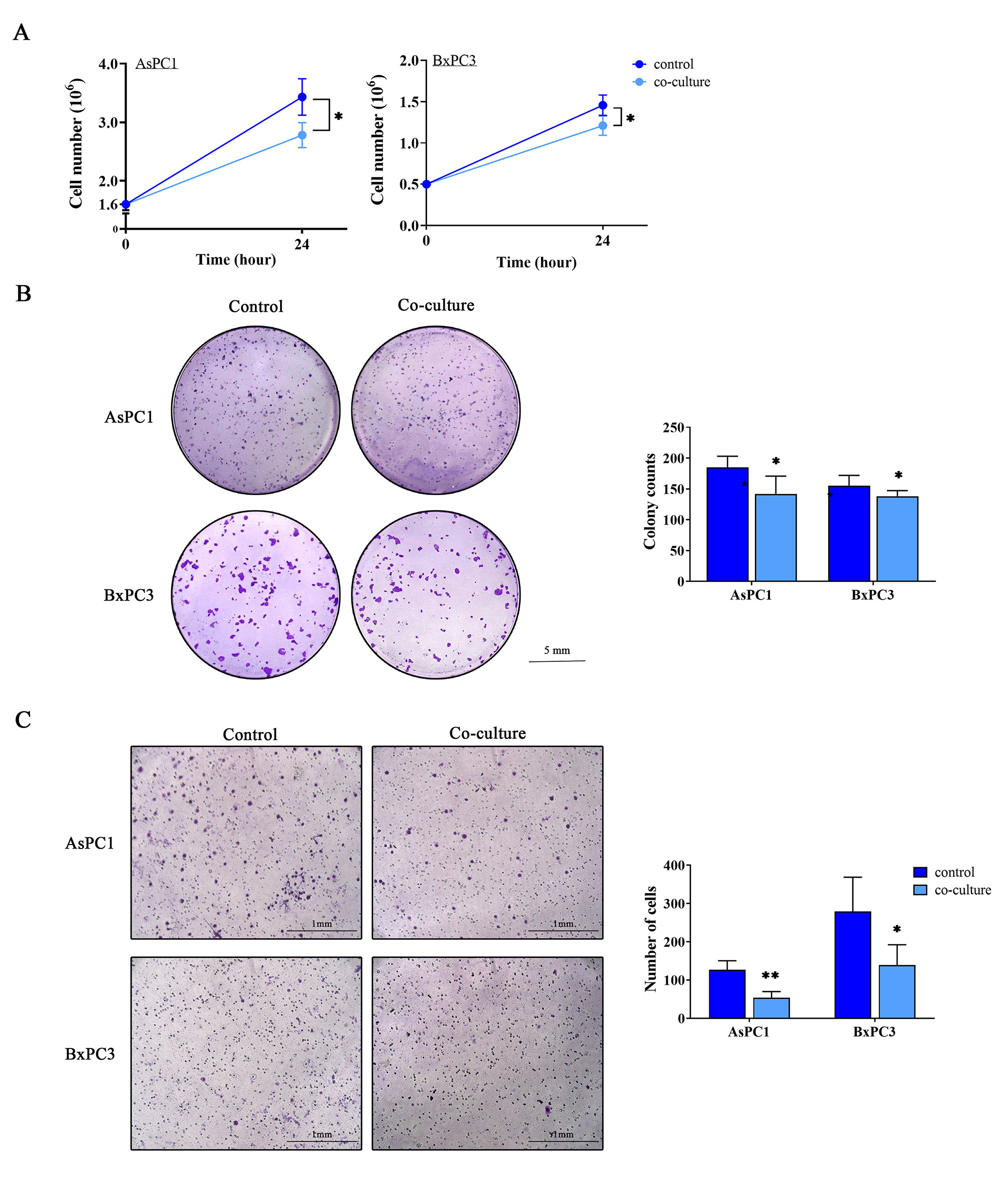

Supplement: Supplementary file 3 — Additional file 3. Figure S2: hAFMSCs suppress cell proliferation in pancreatic cancer cell lines, AsCP1 and BxPC3. (A) Cell numbers of AsPC1 (left) and BxPC3 (middle) were determined by a hemocytometer at 24 h post coculture and quantified (right). (B) Representative images of colony formation of crystal violet-stained cells (left panel) and quantitative statistics (right panel). (C) Represented images were migrated cells stained with crystal violet (left panel) and counted by ImageJ. The quantified data are presented in the right panel. The results are shown as the mean ± SD of 3 independent experiments. * P < 0.05 and ** P < 0.01 vs. the Control group by Student’s t-test. [file 13287_2022_2910_MOESM3_ESM.tif]

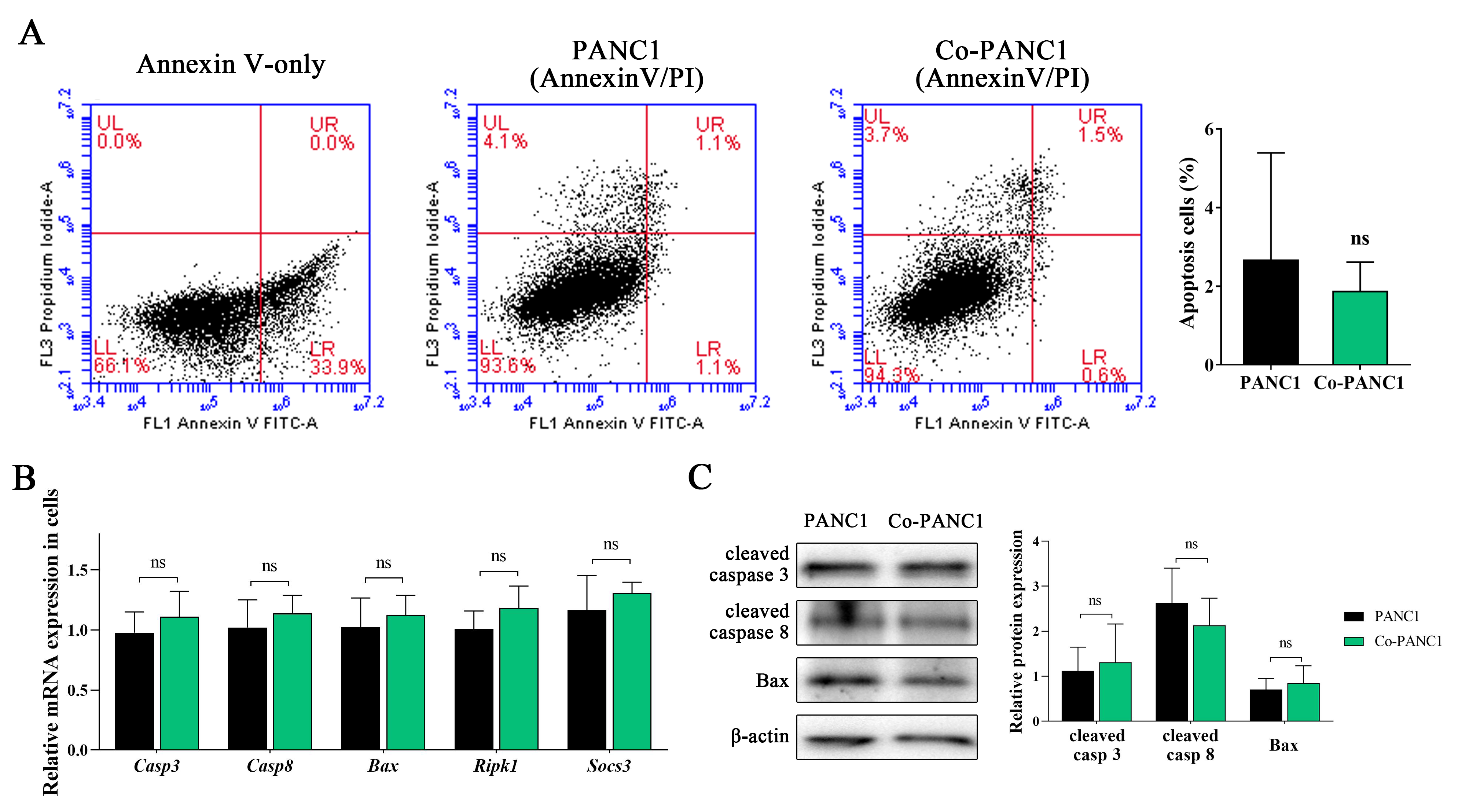

Supplement: Supplementary file 4 — Additional file 4. Figure S3: Cytotoxicity of PANC1 cells cocultured with hAFMSCs. (A) Cells treated with 500 μM hydrogen peroxide for 1 hours, and then stained with annexin V-FITC only. This group was used for setting up the flow cytometric compensation and the set the quadrant gates to separate four populations. After 24 h of coculture, PANC1 and Co-PANC1 cells were assessed by flow cytometric analysis with Annexin V/propidium iodide (PI) staining. The percentages of Annexin V-positive cells (apoptotic cell population) are shown. (B) Relative mRNA expression of pro-apoptotic genes in PANC1 and Co-PANC1 cells. (C) Western blot analysis of pro-apoptotic molecules, including cleaved caspase3/8 and Bax in the cells and quantification is shown in the right panel. Data are presented as the mean ± SD of 3 independent experiments. ns. P > 0.05 vs. the PANC1 group by Student’s t-test. The data are presented as the mean ± SD of 3 independent experiments with 3 technical replicates. [file 13287_2022_2910_MOESM4_ESM.tif]

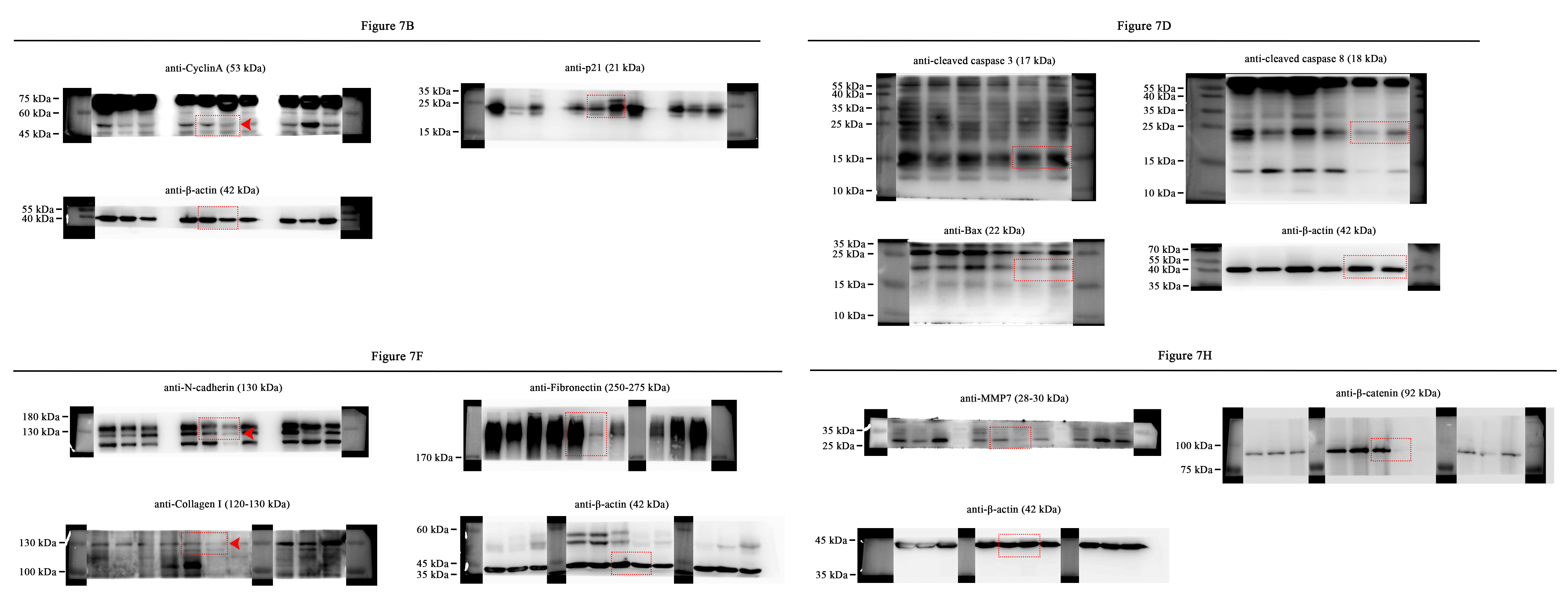

Supplement: Supplementary file 5 — Additional file 5. Figure S4: Original images of Western blots. The original Western blot images for p21 and β-actin are shown in Figure 2C; for cleaved caspase3, cleaved caspase8, Bax, and β-actin are shown in Figure 2E; for N-cadherin, MMP7, and β-actin are shown in Figure 4D. Original images of Western blots. The original Western blot images for CyclinA, p21, and β-actin are shown in Figure 7B; for cleaved caspase3, cleaved caspase8, Bax, and β-actin are shown in Figure 7D; for N-cadherin, fibronectin, collagen I, and β-actin are shown in Figure 7F; for MMP7, β-catenin, and β-actin are shown in Figure 7H. [file 13287_2022_2910_MOESM5_ESM.tif]

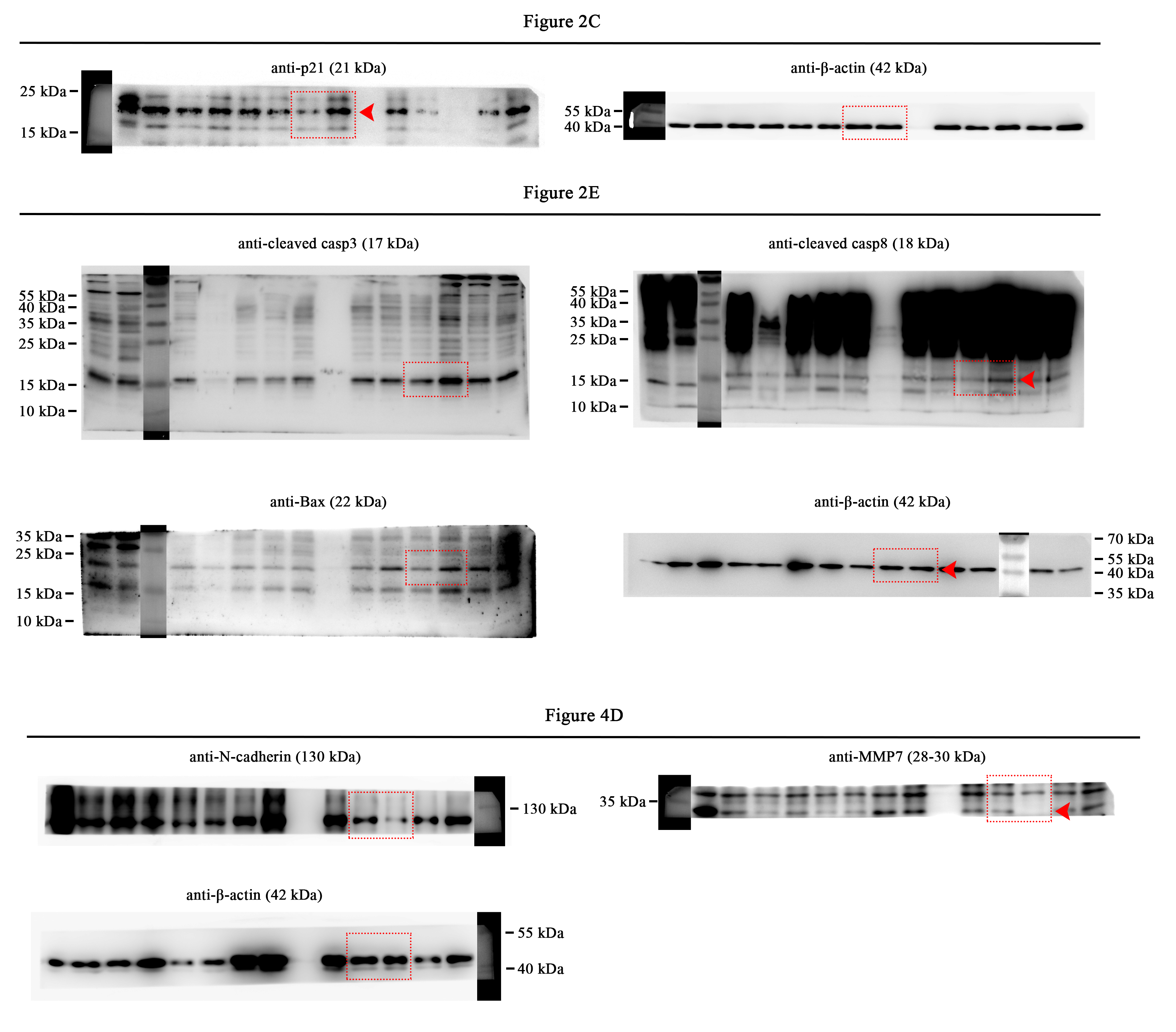

Supplement: Supplementary file 6 — Additional file 6. Figure S4 (cont.): Original images of Western blots. The original Western blot images for CyclinA, p21, and β-actin are shown in Figure 7B; for cleaved caspase3, cleaved caspase8, Bax, and β-actin are shown in Figure 7D; for N-cadherin, fibronectin, collagen I, and β-actin are shown in Figure 7F; for MMP7, β-catenin, and β-actin are shown in Figure 7H. [file 13287_2022_2910_MOESM6_ESM.tif]

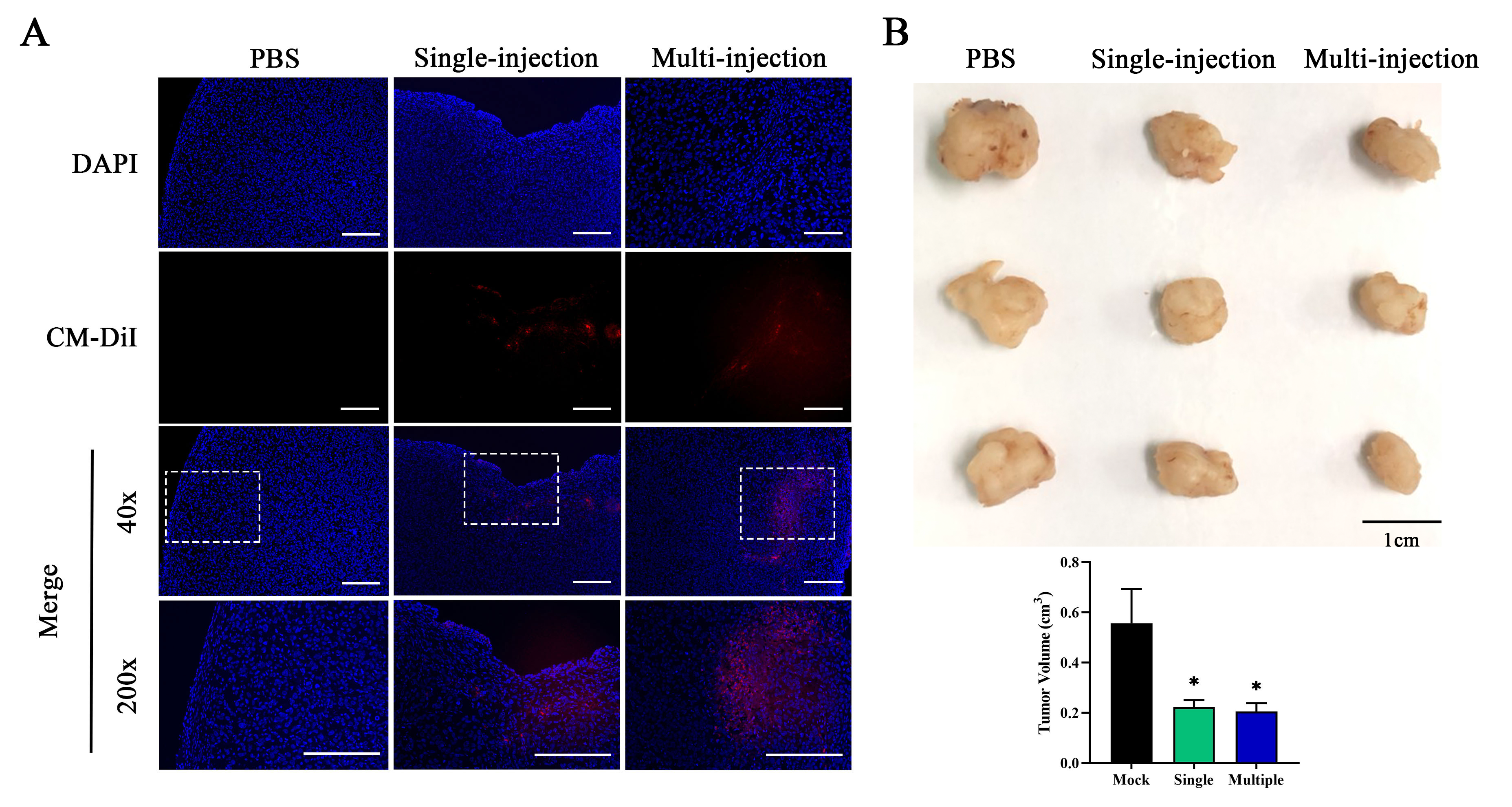

Supplement: Supplementary file 7 — Additional file 7. Figure S5: In vivo CM-Dil-labeled hAFMSC tracing. (A) After four weeks of orthotopic PANC1 cancer cells implantation, mice were randomly divided into single or multiple (once a week) intravenous injection of CM-DiI-stained hAFMSCs (1 × 10^6^ cells in 100 μl PBS/mouse). The recipient mice were maintained for 4 weeks after their first dose of intravenous injection, and then tumors were disassociated from the pancreas. DAPI staining of nuclei (blue). Migrated hAFMSCs located in tumor sites (red). Scale bar = 100 μm. (B upper) Representative images are tumors disassociated from pancreas and aligned with the scale bar of 1 cm. (B bottom) Tumor volumes were calculated with the formula: 1/2 x length x width x depth. Data are demonstrated in mean ± SD, one-way ANOVA followed by Tukey’s post hoc test were performed in comparison with PBS group, *P <0.05. [file 13287_2022_2910_MOESM7_ESM.tif]
