# Supplementary material for: Human amniotic fluid mesenchymal stem cells attenuate pancreatic cancer cell proliferation and tumor growth in an orthotopic xenograft mouse model
Source: Stem Cell Res Ther. 2022 Jun 3;13:235. doi: 10.1186/s13287-022-02910-3 (PMC9166578; doi:10.1186/s13287-022-02910-3)
Supplement: Supplementary file 2 — Additional file 2. Table S1: Oligonucleotide primers used for qRT-PCR analysis in this study. [file 13287_2022_2910_MOESM2_ESM.docx]

**Additional file 2.** Oligonucleotide primers used for qRT-PCR analysis in this study

|  | |  |  |
| --- | --- | --- | --- |
| Gene | Primer sequence | Tm(℃) | Product size |
| *Cyclin A1* | (+) AAGGAGTGTGCGTCAGGACT | 53.8 | 177 |
|  | (‒) ACCCTGTAAATGCAGCAAGG | 51.8 |  |
|  |  |  |  |
| *Cyclin A2* | (+) GGATGGTAGTTTTGAGTCACCAC | 55.3 | 202 |
|  | (‒) CACGAGGATAGCTCTCATACTGT | 55.3 |  |
|  |  |  |  |
| *Cyclin B1* | (+) CATGGTGCACTTTCCTCCTT | 51.8 | 145 |
|  | (‒) CAGGTGCTGCATAACTGGAA | 51.8 |  |
|  |  |  |  |
| *p21* | (+) TGTCACTGTCTTGTACCCTTG | 52.4 | 228 |
|  | (‒) GGCGTTTGGAGTGGTAGAA | 51.1 |  |
|  |  |  |  |
| *N-cadherin* | (+) TCAAAGCCTGGAACATATGTGAT | 51.7 | 332 |
|  | (‒) TCACCATAAAACGTCATGGCA | 50.5 |  |
|  |  |  |  |
| *Vimentin* | (+) TCTACGAGGAGGAGATGCGG | 55.9 | 194 |
|  | (‒) GGTCAAGACGTGCCAGAGAC | 55.9 |  |
|  |  |  |  |
| *Fibronectin* | (+) CCCACCGTCTCAACATGCTTAG | 56.7 | 264 |
|  | (‒) CTCGGCTTCCTCCATAACAAGTAC | 57.4 |  |
|  |  |  |  |
| *Collagen I* | (+) CGGAGGAGAGTCAGGAAGG | 55.4 | 111 |
|  | (‒) CACAAGGAACAGAACAGAACAG | 53 |  |
|  |  |  |  |
| *HIF-1α* | (+) CACCACAGGACAGTACAGGAT | 54.4 | 146 |
|  | (‒) CGTGCTGAATAATACCACTCACA | 53.5 |  |
|  |  |  |  |
| *SMAD4* | (+) ACGAACGAGTTGTATCACCTGG | 54.8 | 173 |
|  | (‒) TGCACGATTACTTGGTGGATG | 52.4 |  |
|  |  |  |  |
| *LRP* | (+) CGCAACGTCTGCAAGTTCG | 53.2 | 123 |
|  | (‒) TGGAGGCGCATCCTTTTCC | 53.2 |  |
|  |  |  |  |
| *ZEB1* | (+) TTACACCTTTGCATACAGAACCC | 53.5 | 100 |
|  | (‒) TTTACGATTACACCCAGACTGC | 53 |  |
|  |  |  |  |
| *MMP7* | (+) GAGTGAGCTACAGTGGGAACA | 54.4 | 158 |
|  | (‒) CTATGACGCGGGAGTTTAACAT | 53 |  |
|  |  |  |  |
| *Collagen IV* | (+) AGATAAGGGTCCAACTGGTGT | 52.4 | 232 |
|  | (‒) ACCTTTAACGGCACCTAAAATGA | 51.7 |  |
|  |  |  |  |
| *Laminin β1* | (+) TGACTTTCAAGACATTCCGTCC | 53 | 90 |
|  | (‒) AGGCGAAGTATCTATACACACCC | 55.3 |  |
|  |  |  |  |
| *β-catenin* | (+) CTTCTGCGCGACTTATAAGA | 49.7 | 258 |
|  | (‒) ATTGTCCACGCTGGATTTTC | 49.7 |  |
|  |  |  |  |
| *Caspase 3* | (+) CATGGAAGCGAATCAATGGACT | 53.0 | 139 |
|  | (‒) CTGTACCAGACCGAGATGTCA | 54.4 |  |
|  |  |  |  |
| *Caspase 8* | (+) GTTGTGTGGGGTAATGACAATCT | 53.5 | 222 |
|  | (‒) TCAAAGGTCGTGGTCAAAGC | 51.8 |  |
|  |  |  |  |
| *Caspase 9* | (+) CTGTCTACGGCACAGATGGAT | 54.4 | 177 |
|  | (‒) GGGACTCGTCTTCAGGGGAA | 55.9 |  |
|  |  |  |  |
| *BAX* | (+) GCC CTT TTG CTT CAG GGT TT | 51.8 | 356 |
|  | (‒) TCC AAT GTC CAG CCC ATG AT | 51.8 |  |
|  |  |  |  |
| *RIPK1* | (+) TATCCCAGTGCCTGAGACCAAC | 56.7 | 127 |
|  | (‒) GTAGGCTCCAATCTGAATGCCAG | 57.1 |  |
|  |  |  |  |
| *SOCS3* | (+) AAGGCTCCTTTGTGGACTTCA | 52.4 | 244 |
|  | (‒) AAACTTGCTGTGGGTGACCAT | 52.4 |  |
|  |  |  |  |
| *β-actin* | (+) GCGAGAAGATGACCCAGATC | 53.8 | 103 |
|  | (‒) CCAGTGGTACGGCCAGAGG | 57.6 |  |
